# Single‐cell atlas reveals heterogeneous response to FcRn blockade in anti‐AChR antibody‐positive generalised myasthenia gravis

**DOI:** 10.1002/ctm2.70436

**Published:** 2025-08-06

**Authors:** Hui‐Ning Li, Jingjing Liu, Xiao‐Yu Huang, Lijie Zhu, Zhirui Liu, Chun‐Sheng Yang, Bo Zhang, Shixiong Huang, Fu‐Dong Shi, Zhigang Cai, Chao Zhang

**Affiliations:** ^1^ Department of Neurology Tianjin Neurological Institute Tianjin Medical University General Hospital Tianjin China; ^2^ Tianjin Key Laboratory of Inflammatory Biology Department of Pharmacology School of Basic Medicine Tianjin Medical University Tianjin China; ^3^ State Key Laboratory of Experimental Hematology National Clinical Research Center for Blood Diseases, Haihe Laboratory of Cell Ecosystem Tianjin China; ^4^ Province and Ministry Co‐Sponsored Collaborative Innovation Center for Medical Epigenetics, Department of Bioinformatics School of Basic Medicine Tianjin Medical University Tianjin China; ^5^ Department of Neurology Hainan General Hospital Hainan Affiliated Hospital of Hainan Medical University Haikou China; ^6^ Department of Neurology National Clinical Research Center for Neurological Diseases of China Beijing Tiantan Hospital Capital Medical University Beijing China

**Keywords:** FcRn blockade, myasthenia gravis, single‐cell RNA sequencing, therapeutic response

## Abstract

**Background:**

Myasthenia gravis (MG) is an autoimmune disease predominantly driven by autoantibodies targeting acetylcholine receptor (AChR), resulting in muscle weakness. Efgartigimod, a neonatal Fc receptor (FcRn) blocker, reduces pathogenic immunoglobulin G in anti‐AChR antibody‐positive generalised MG (gMG). This study aimed to identify immune mechanisms underlying MG pathology and response to efgartigimod.

**Methods:**

We constructed a single‐cell atlas of peripheral immune cells from treatment‐naïve and efgartigimod‐treated patients with gMG. Comprehensive immunophenotyping was performed to compare the clonal diversity of B‐ and T‐cell populations, alongside experimental validation to assess the activation of Th17‐related pathways before and after FcRn blockade.

**Results:**

B cells in patients with gMG exhibit heightened activation and differentiation, while T cells display distinct pro‐inflammatory phenotypes. Enhanced intercellular signalling contributed to the pathogenicity associated with gMG. Efgartigimod mitigated upregulated antigen processing and presentation pathways in MG. Additionally, B‐cell clonal diversity and IGHG1‐bearing B‐cell receptors increased. Transcriptional factor alterations were noted in suboptimal responders. Regulation of T‐cell activity, particularly within Th17‐related pathways, was associated with remission rates.

**Conclusions:**

These findings underscore immune heterogeneity and dynamics during efgartigimod treatment, providing mechanistic insights into therapeutic response in gMG.

**Key points:**

- Aberrant B cells and pro‐inflammatory T cells contribute critically to generalised myasthenia gravis (gMG) pathogenesis.
- Neonatal Fc receptor (FcRn) blockade induces immunoglobulin G (IgG) depletion feedback, reflected by increased class‐switched BCRs.
- Th17 cell proliferation is attenuated following FcRn blockade.
- Antigen processing and presentation pathways are downregulated after FcRn blockade in gMG.

## Graphical abstract

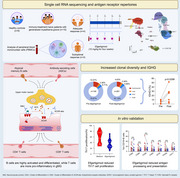

## 1 BACKGROUND

Myasthenia gravis (MG) is an autoimmune disorder characterised by skeletal muscle weakness resulting from impaired neurotransmission at the neuromuscular junction.^1^ Approximately 80%–85% of individuals with generalised MG (gMG) possess pathogenic antibodies targeting the acetylcholine receptor (AChR‐abs).^2^ These antibodies promote internalisation and degradation of AChR and activate the complement system. Aberrant B‐cell activity plays a crucial role in MG pathogenesis, including antigen presentation, production of AChR‐abs and secretion of pro‐inflammatory cytokines. CD4^+^ T cells are essential for supporting B‐cell‐driven autoimmunity in MG.^3^ Additionally, AChR‐abs may activate myeloid cells and foster a pro‐inflammatory milieu, which enhances the crosstalk between autoreactive T and B cells. However, the integrated immune landscape in gMG remains elusive.^4^ Single‐cell RNA sequencing (scRNA‐seq) could be a powerful approach for profiling the immune repertoire in autoimmune diseases, including MG.^5^, ^6^

In MG, most AChR‐abs produced by B cells belong to the immunoglobulin G1 (IgG1) and IgG3 subclasses, with a dynamic process mediated by the neonatal Fc receptor (FcRn), which regulates IgG trafficking and recycling in humoral immunity.^7^ FcRn also facilitates the internalisation and trafficking of antigen‐bound IgG immune complexes into compartments for degradation and antigen presentation by antigen‐presenting cells.^8^, ^9^ Thus, FcRn supports the sustained presence of IgG autoantibodies in IgG‐mediated diseases. Efgartigimod, a human IgG1 antibody Fc fragment, competitively binds to FcRn and accelerates lysosomal degradation of IgG, thereby reducing serum IgG levels.^10^ Efgartigimod has shown efficacy and safety in patients with gMG.^11^ However, only about two‐thirds of patients benefit adequately from efgartigimod, while up to 30% show a suboptimal response.^11^ The mechanisms underlying the heterogeneous response to FcRn blockade remain unclear. This study aimed to elucidate the molecular changes associated with FcRn blockade by efgartigimod by examining immune landscapes in adult patients with gMG exhibiting varying clinical responses.

## 2 METHODS

### 2.1 Study design and recruitment

**FIGURE 1:**
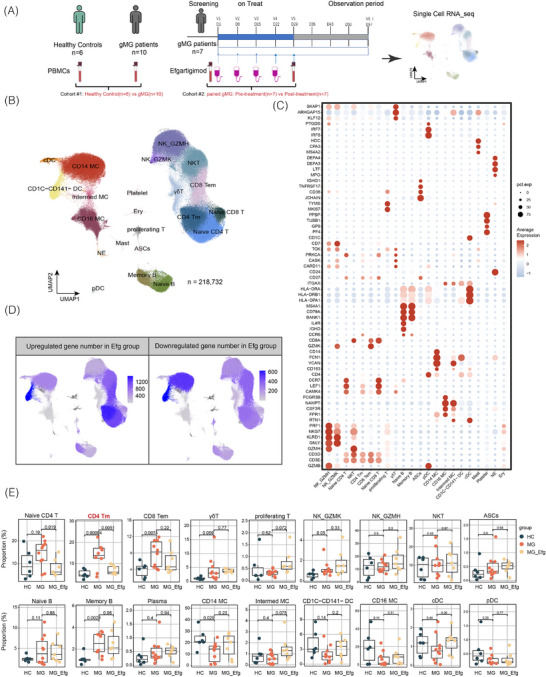
Peripheral immune landscapes in generalised myasthenia gravis (gMG). (A) Flowchart illustrating the study design, including single‐cell RNA sequencing (scRNA‐seq) analysis of immune repertoires in peripheral blood mononuclear cells (PBMCs) from healthy controls (HCs) and patients with gMG. Cohort #1 consisted of 10 immuno‐treatment‐naïve patients with gMG and six HCs, while cohort #2 comprised paired PBMC samples from seven patients with gMG before and after neonatal Fc receptor (FcRn) blockade treatment (four weekly infusions of efgartigimod [Efg], 10 mg/kg), using single‐cell transcriptomics and T/B‐cell receptor immune repertoire profiling. Post‐treatment samples were collected 1 week after the fourth Efg infusion (V5). If receiving corticosteroids and/or nonsteroidal immunosuppressive therapies (NSISTs), must be on a stable dose for ≥1 month prior to screening. (B) Uniform manifold approximation and projection (UMAP) of scRNA‐seq datasets showing 22 immune cell clusters across PBMCs from 10 patients with gMG and six HCs (*n* = 218 732 cells). (C) Bubble heatmap displaying expression levels of marker genes across identified cell type clusters. (D) UMAP plot indicating the number of upregulated and downregulated genes across PBMC clusters after FcRn blockade with Efg. (E) Boxplots showing immune cell cluster frequencies in HCs (*n* = 6), treatment‐naïve patients with gMG (*n* = 10) and patients treated with FcRn blockade (*n* = 7). Student's *t*‐test *p‐*values are shown for indicated comparisons.

The study flowchart is presented in Figure 1A. Cohort #1 consisted of 10 immuno‐treatment‐naïve patients with gMG and six healthy controls (HCs). Cohort #2 comprised paired peripheral blood mononuclear cell (PBMC) samples from seven patients with gMG collected before and after FcRn blockade treatment (four weekly infusions of efgartigimod, 10 mg/kg). Post‐treatment samples were obtained 1 week after the fourth efgartigimod infusion (V5, Figure 1A). Patients receiving concomitant gMG therapies were required to be on a stable dose for at least 1 month prior to enrollment and to maintain this regimen throughout the study. Exclusion criteria included prior exposure to monoclonal antibody‐based biological therapies or receipt of intravenous immunoglobulin or plasma exchange within 1 month before enrollment. Cohort #3 consisted of PBMC samples collected from newly diagnosed, treatment‐naïve MG patients for in vitro experiments. The diagnosis of gMG was confirmed based on fluctuating generalised skeletal muscle weakness and the detection of serum AChR‐abs with at least one of the following criteria: (1) a positive neostigmine test or (2) abnormal results from repetitive nerve stimulation testing.^1^ Clinical assessments, including the Myasthenia Gravis Activities of Daily Living (MG‐ADL) scale and the Quantitative Myasthenia Gravis (QMG) score, were performed weekly. An adequate treatment response was defined as an improvement of more than three points in the MG‐ADL scale at the final evaluation compared to baseline, exceeding the standard clinically meaningful change of a two‐point reduction, and thereby reflecting a more stringent criterion.^12^

### 2.2 Library construction and sequencing of human scRNA‐seq

Peripheral blood samples were collected using the 10× Chromium System (10× Genomics, Pleasanton). Single cells were encapsulated into droplets according to the manufacturer's protocol, and the cDNA library was prepared and sequenced using a HiSeq PE150 (Illumina) at Novogene. For scRNA‐seq library construction, the SeekOne Digital Droplet Single Cell 3′ Library Preparation Kit (Proteigene) was used, following these steps: (1) approximately 12 000 PBMCs were mixed with reverse transcription reagents and loaded into SeekOne chip sample wells; barcode hydrogel beads and partitioning oil were added to the corresponding wells; (2) droplets were formed, reverse transcription was conducted and inactivation was performed; (3) cDNA was purified and amplified by PCR; (4) the amplified cDNA was cleaned, fragmented, end‐repaired, A‐tailed and ligated to sequencing adapters; (5) the gene 3′ polyA region was amplified by indexed PCR, incorporating the cell barcode and Unique Molecular Identifier; and (6) the final cDNA product was purified using the SPRIselect Reagent Kit (Beckman Coulter). A NovaSeq 6000 (Illumina) was utilised for second‐generation sequencing with a PE150 read length.

### 2.3 scRNA‐seq data processing

The reads of each gene in the scRNA‐seq sample were quantified against the GRCh38 human reference genome using the Cell Ranger software suite (version 8.0, 10× Genomics). Seurat objects were created using Seurat 4.4.0, with quality control criteria including cells with gene expression exceeding 200 but below 7500 and mitochondrial gene proportions under 20%. After filtering, 96 262 cells and 27 930 genes were retained from the human samples. Data normalisation was performed using the NormaliseData function, and high‐variance genes were identified with FindVariableFeatures. *Z*‐score transformation was applied via ScaleData for Principal Component Analysis (PCA)‐based dimensionality reduction. Batch effects were corrected using the RunHarmony function. Cell clustering was conducted with the FindNeighbors and FindClusters functions. Non‐linear dimensionality reduction and visualisation were achieved via run uniform manifold approximation and projection (RunUMAP). To integrate datasets from different batches for unsupervised clustering, the Harmony algorithm corrected for batch effects. Highly expressed genes in each cluster were identified with FindAllMarkers, and top‐ranked genes were designated as cluster‐specific markers. Cell types were annotated based on canonical marker gene.

### 2.4 Differentially expressed genes and pathway enrichment

Differentially expressed genes (DEGs) between the compared groups were identified using the FindMarkers function with the parameters ‘min.pct’ = .25 and ‘logfc.threshold’ = .25. Gene set variation analysis (GSVA)‐based pathway enrichment was subsequently performed on the DEGs using the C2 gene set collection.

### 2.5 Gene set scoring

Gene set scores for each cell were estimated using the AddModuleScore function in Seurat. This function calculates the average expression of a defined gene program at the single‐cell level, while subtracting the aggregated expression of randomly selected control feature sets. Genes were binned based on average expression, and control features were randomly selected from each bin. Details of all gene sets are provided in Table S2.

### 2.6 Cell‒cell communication analysis

The interaction intensity of information flow between cells was calculated using CellChat (version 1.6.1). To infer cell state‐specific communication, CellChat identified differentially expressed signalling genes across all cell populations in the scRNA‐seq dataset using the Wilcoxon rank‐sum test with a significance threshold of  .05. Ligand‒receptor‐mediated signalling interactions were modelled based on the law of mass action. Communication within specified subsets was predicted using NicheNet (version 2.0.4), which assessed target genes and ligands with high activity rankings and inferred ligand‐to‐target signal transduction pathways.

### 2.7 Cell development analysis

Cell development was analysed using Monocle (version 2.26.0). Monocle employs a pseudotime ordering strategy to place individual cells along a trajectory reflecting a biological process, such as cell differentiation, by utilising asynchronous cell progression through these processes. CytoTRACE (version 0.3.3) assessed the differentiation status of cells based on clustering results from RunUMAP. The differentiation state, captured by the gene count signature, predicted the differentiation potential of cells based on key RNA characteristics derived from scRNA‐seq data.

### 2.8 Transcriptional factor analysis

Gene regulatory network (GRN) inference and regulon activity quantification were performed using pySCENIC (v0.12.1) following the standard pipeline. scRNA‐seq count matrices were preprocessed to retain high‐quality cells and variable genes. First, co‐expression modules were inferred using GRNBoost2 (implemented in pySCENIC). Next, cis‐regulatory motif enrichment analysis was conducted for each module using the RcisTarget database. Modules with significant motif enrichment (normalised enrichment score > 3.0; AUC threshold > .005) were retained as regulons. Finally, AUCell was applied to calculate regulon activity scores per cell, representing the enrichment of regulon target genes within each cell's ranked expression profile.

### 2.9 TCR and BCR repertoire analysis

T cell receptor (TCR) and B cell receptor (BCR) repertoire analyses were performed using the scRepertoire R package (v1.8.0), integrated with single‐cell transcriptomic data processed via Seurat. For TCR analysis, paired α and β chain contig annotations were imported, and merged into the Seurat object using shared cell barcodes. Non‐productive TCR sequences were filtered out, and cells with more than two TCR chains were excluded to avoid potential multiplets. Similarly, for BCR analysis, only cells with productive immunoglobulin heavy (IGH) and light (IGK/IGL) chain pairs were retained, and those containing more than one light chain pair were removed. Clonotypes were defined based on the CDR3 amino acid sequences of paired chains. Clonal expansion was evaluated by quantifying the abundance and frequency of cells within each unique clonotype. All visualisations and downstream statistical analyses were performed using built‐in functions in scRepertoire and ggplot2.

### 2.10 Flow cytometry assays

PBMCs from patients with gMG were isolated from fresh anticoagulated blood using Ficoll gradient separation. They were collected and stained with antibody cocktails to assess B‐cell antigen presentation. For intracellular cytokine staining, PBMCs were treated with 2 µL of eBioscience Cell Stimulation Cocktail for 5 h (Thermo Fisher Scientific). Cells were then surface‐stained with anti‐CD3‐FITC and anti‐CD4‐BV421, fixed and permeabilised using the eBioscience Intracellular Fixation & Permeabilisation Buffer Set (Thermo Fisher Scientific), and stained with anti‐IL‐2‐PE, anti‐IL‐4‐PE‐Cy7, anti‐IL‐10‐Percp‐Cy5.5, anti‐TNF‐α‐APC‐Cy7, anti‐IFN‐γ‐Percp‐Cy5.5, anti‐IL‐6‐PE‐Cy7 and anti‐IL‐17A‐PE. Samples were analysed on a FACSAria III flow cytometer (BD Biosciences), and data were processed using FlowJo v10.0.7. The detailed list of key resources is provided in Table S1. The gating strategies are shown in Figure S1A‒C.

### 2.11 Proliferation of Th17 cells was detected in vitro

To assess the effect of FcRn blockade on the differentiation and proliferation of Th17 cells, PBMCs were labelled with 5 µM Carboxyfluorescein Succinimidyl Ester (CFSE) for 20 min at 37°C, washed and resuspended in culture medium. Subsequently,  .5‒1 million cells per well were stimulated with anti‐CD3 (2 µg/mL) and anti‐CD28 (4 µg/mL) monoclonal antibodies in the presence or absence of efgartigimod (500 µg/mL) and cultured in 24‐well plates for 3 days. The selected efgartigimod concentration used here was based on dose‒response optimisation (Figure S1D,E). For intracellular cytokine staining, the cultured PBMCs were treated with 2 µL of eBioscience Cell Stimulation Cocktail (Thermo Fisher Scientific) for 5 h. Cells were then surface‐stained with anti‐CD3‐FITC and anti‐CD4‐BV421, fixed and permeabilised using the eBioscience Intracellular Fixation & Permeabilisation Buffer Set (Thermo Fisher Scientific), and stained with anti‐IL‐17A‐PE‐Cy7. Samples were analysed as described above. The detailed list of key resources is provided in Table S1.

### 2.12 Quantification and statistical analysis

Bioinformatic and statistical analyses are described in the relevant sections of the main text and summarised in Table S1, with all analyses performed using R (v3.5.1, v4.3.0; R Foundation). Experimental data are presented as mean ± standard error of the mean (SEM). Paired *t*‐tests were used to compare cell subclusters in vitro experiments before and after treatment.

## 3 RESULTS

### 3.1 FcRn blockade alters the landscapes of PBMCs in patients with gMG

To assess immune changes in patients with gMG, scRNA‐seq of PBMCs from 16 samples was performed to generate high‐quality transcriptomes by comparing patients with gMG to HCs (Figure 1A). Ten treatment‐naïve patients with AChR‐ab‐positive gMG were recruited as Cohort #1 (mean age: 60.0 ± 11.9 years; 60% female; Table S3). The median MG‐QMG score was 22 (range 13–26), and the median MG‐ADL score was 9 (range 4–13). Disease severity was classified as Myasthenia Gravis Foundation of America (MGFA) class II (40%), class III (20%) and class IV (40%). Seven patients with AChR‐ab‐positive gMG received weekly efgartigimod infusions (10 mg/kg) for 4 weeks (one cycle) as Cohort #2 (pre‐treatment vs. post‐treatment). This cohort had a mean age of 60.3 ± 15.2 years, with 29% female (Table S3). At baseline, the median MG‐QMG score was 20 (range: 14–23), and the median MG‐ADL score was 9 (range: 8–13). The MGFA classification distribution was as follows: class II (43%), class III (43%) and class IV (14%).

Using canonical markers, 22 immune cell clusters were identified: T cells (*CD3D*, *CD3E*), further divided into naïve CD4^+^ T cells, CD4^+^ memory T (Tm) cells, naïve CD8^+^ T cells, CD8^+^ effector memory T (Tem) cells and proliferating T cells (*TYMS*, *MKI67*); B cells (*CD79A*) stratified into naïve B cells (*MS4A1*, *IGHD*), memory B cells (*MS4A1*, *BANK1*) and antibody‐secreting cells (ASCs) (*CD27*, *CD38*, *TNFRSF17*, *IGHG1*); innate immune cells, such as γδ T cells and NK cells (*NKG7*), with NK cells categorised into NK_*GZMH*, NK_*GZMK* and NKT subsets; dendritic cells (DCs, *HLA‐DRA*, *HLA‐DRB1*) classified as *CD1C^−^ CD141^−^* DC, conventional DC and plasmacytoid DC; monocytes (*CD14*, *FCGR3A*) divided into *CD14* ^+^ monocytes, intermediate monocytes and *CD16* ^+^ monocytes; neutrophils, mast cells, erythrocytes and platelets (Figure 1B,C). Next, DEGs across clusters, before and after FcRn blockade with efgartigimod, were visualised using UMAP (Figure 1D). Following treatment, upregulated DEGs were predominantly localised to T cells and DCs, whereas downregulated DEGs were primarily observed in monocytes and DCs (Figure 1D).

Analysis of immune cell revealed significantly higher frequencies of memory B cells, CD4^+^ Tm cells and CD8^+^ Tem cells in patients with gMG compared to HCs. Among these subsets, only CD4^+^ Tm cells showed a marked reduction in frequency after efgartigimod treatment (Figure 1E).

### 3.2 Cells are highly activated and differentiated in gMG

**FIGURE 2:**
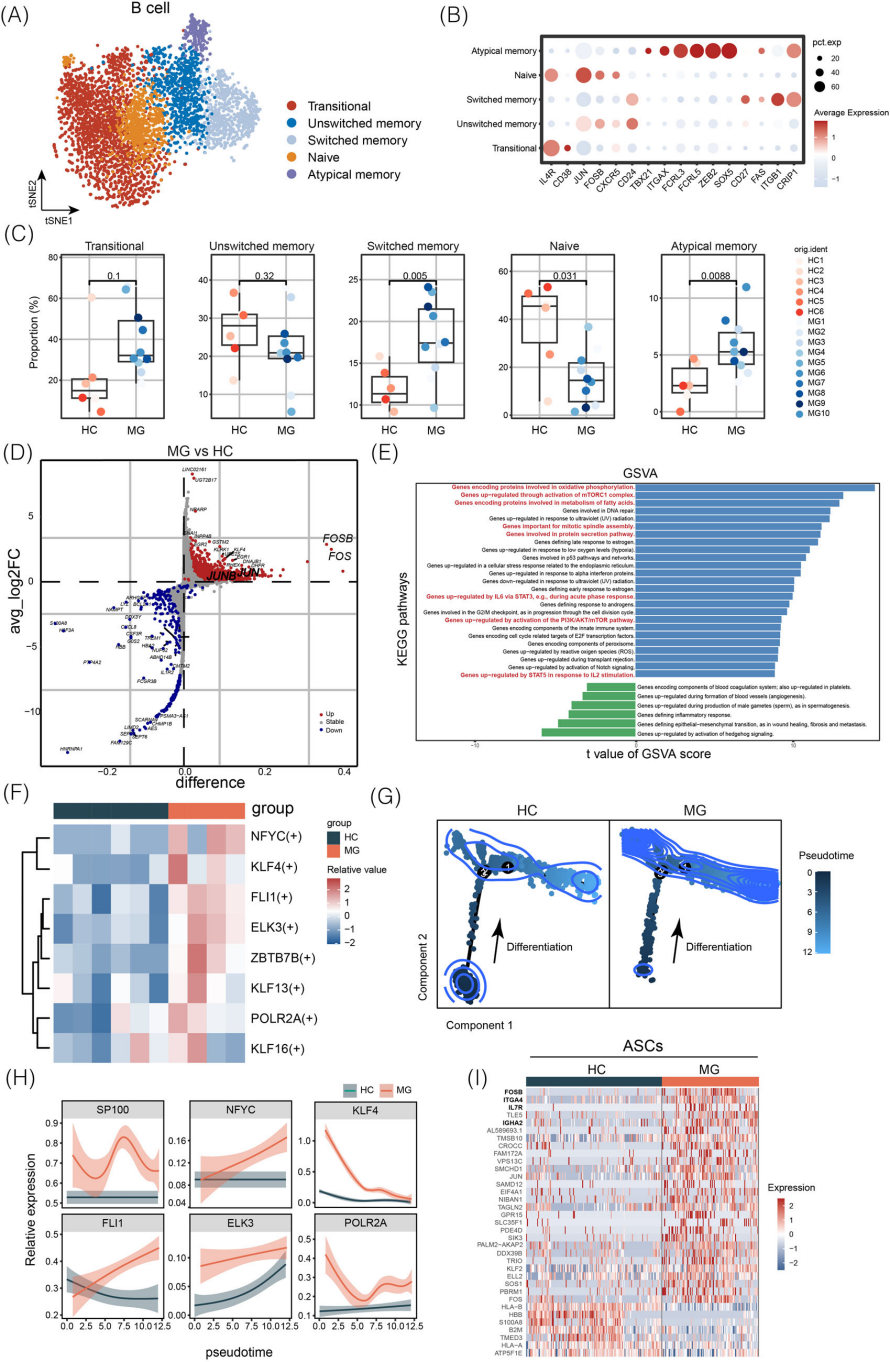
B‐cell abnormalities in generalised myasthenia gravis (gMG). (A) Uniform manifold approximation and projection (UMAP) plot showing five B‐cell subtypes from healthy controls (HCs) and patients with gMG. Subtypes include naïve, transitional, unswitched memory, switched memory and atypical memory B cells. (B) Expression levels of marker genes across B‐cell subclusters. (C) Boxplots comparing proportions of B‐cell subclusters between HCs and patients with gMG. *p*‐Values are shown for indicated comparisons. (D) Volcano plot of differentially expressed genes (DEGs) in B cells from patients with gMG compared to HCs. (E) Gene set variation analysis (GSVA) enrichment of hallmark gene sets in B cells from patients with gMG versus HCs, with T values calculated by limma regression. (F) Heatmap of transcription factor activity in B cells from HC and gMG groups. (G) B‐cell development trajectories in HCs and patients with gMG, with colours indicating cell states from primitive (dark blue) to mature (light blue). (H) Trends in the expression of selected genes across B‐cell development trajectories. (I) Top DEGs in antibody‐secreting cells (ASCs) from HC and gMG groups.

We further profiled naïve and memory B cells in Figure 1B into five subsets (Figure 2A): naïve B cells (*IL4R*, *IGHD*), transitional B cells (*IL4R*, *CD38*), unswitched memory B cells (*CD24*, *IGHA1*), switched memory B cells (*ITGB1*, *IGHG1*) and atypical memory B cells (age‐associated B cells, *ITGAX*, *ZEB2*, *TBX21*) (Figure 2B). Importantly, switched memory and atypical memory B cells were expanded in patients with gMG compared to those in HCs (Figure 2C). DEG analysis revealed marked upregulation of genes involved in B‐cell activation and migration in gMG, including *FOS*, *FOSB*, *CD52*, *HLA‐DQB1* and *ITGA4* (Figure 2D and Table S4). Naïve B cells displayed increased expression of *IL6* and *NR4A1* and reduced expression of *CD40LG* and *IL2RB*. Switched memory B cells exhibited elevated expression of *RGS1*, *MAL*, *CCL3L1* and *TNFSF9*, whereas atypical memory B cells uniquely expressed high levels of *FCRL5* (but not *FCRL4*) with reduced *SMURF1* (Table S5).

In the GSVA, upregulated genes in gMG B cells were associated with overactivated pathways, including oxidative phosphorylation, PI3K/AKT/mTOR, mTORC1 activation, fatty acid metabolism, mitotic spindle assembly, protein secretion, and IL‐6/STAT3 and IL‐2/STAT5 signalling, suggesting enhanced proliferation and survival capacity (Figure 2E). Transcription factor (TF) analysis revealed significantly higher expression of *KLF4*, *FLI1*, *ELK3* and *ZBTB7B* in MG B cells (Figure 2F). Pseudotime analysis using DDRTree identified an activated naïve B‐cell state and increased differentiation towards mature phenotypes in patients with gMG (Figure 2G). Key differentiation‐associated TFs, including *SP100*, *NFYC*, *KLF4* and *POLR2A*, exhibited distinct upregulation patterns along the B‐cell trajectory (Figure 2H). Given the critical role of ASCs in producing high‐affinity antibodies, we examined transcriptional differences between ASCs from patients with gMG and HCs. As expected, these differences were pronounced, with elevated expression of *FOSB*, *ITGA4*, *IL7R* and *IGHA2* (Figure 2I and Table S6). These findings highlight the highly activated and differentiated state of B cells in patients with gMG compared to HCs.

### 3.3 Cells are more pro‐inflammatory in gMG

**FIGURE 3:**
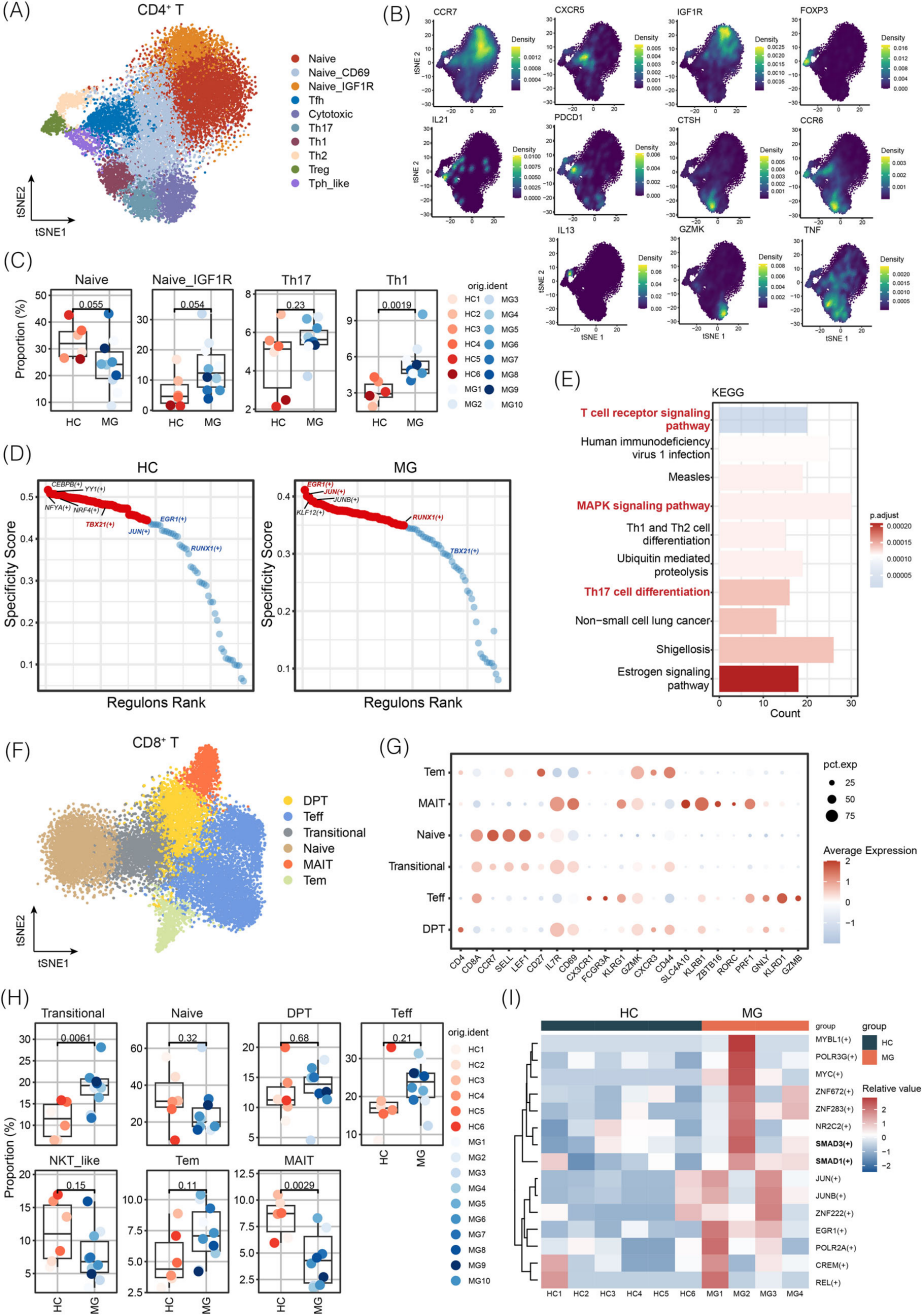
T‐cell abnormalities in generalised myasthenia gravis (gMG). (A) Uniform manifold approximation and projection (UMAP) plot of 10 CD4^+^ T‐cell subtypes from healthy controls (HCs) and patients with gMG. (B) Expression levels of marker genes across CD4^+^ T‐cell subclusters. (C) Boxplots showing the proportions of CD4^+^ T‐cell subclusters in HCs and patients with gMG. *p*‐Values are indicated for comparisons. (D) Transcription factor activity ranked in CD4^+^ T cells from HC and gMG groups. (E) Kyoto Encyclopaedia of Genes and Genomes pathway enrichment analysis of upregulated genes in CD4^+^ T cells from patients with gMG. (F) Uniform manifold approximation and projection (UMAP) plot of six CD8^+^ T‐cell subtypes in HCs and patients with gMG. (G) Expression levels of marker genes across CD8^+^ T‐cell subclusters. (H) Boxplots comparing proportions of CD8^+^ T‐cell subclusters between HCs and patients with gMG. *p*‐Values are indicated for comparisons. (I) Ranked transcription factor regulon activity in CD8^+^ T cells from HC and gMG groups.

Ten subsets of CD4^+^ T cells in patients with gMG were identified (Figure 3A). Among these, three populations were categorised as naïve subsets based on higher expression of *CCR7* and *CD6*9 (Figure S2). Five clusters corresponded to T helper (Th) cell subtypes, profiled as T follicular helper (Tfh) (*CXCR5*), Tph‐like (*PDCD1*), Th1 (*IL2*), Th2 (*IL13*) and Th17 (*CXCR6* and *CTSH*) subsets (Figure 3B). Regulatory T cells were marked by high *FOXP3* expression, and a distinct cluster of CD4*^+^* T cells expressed *GZMK*, indicative of a cytotoxic signature (Figure 3B). Th1 and Th17 subsets exhibited strong expression of *TNF‐α*, whereas *IL21* was mounted in Tph‐like cells, indicating a more pro‐inflammatory state in these subsets (Figure 3B). The proportion of Th1 cells was significantly elevated in gMG (Figure 3C). Meanwhile, there was a decreasing trend in naïve T cells; however, naïve T cells with high *IGF1R* expression tended to increase. Activation of *IGF1R* can enhance Th17 cell differentiation and IL‐17 production.^13^ Next, TF analysis in patients with gMG revealed elevated activities of *JUN*, *EGR1* and *RUNX1* in MG (Figure 3D). Notably, *RUNX1* positively regulates *RORC* expression in Th17 cells. Conversely, activation of *TBX21*, a TF that initiates Th1 differentiation by activating Th1‐specific gene programs while repressing Th2 and Th17 programs, was reduced in patients with gMG, suggesting a potential shift towards Th2 and Th17 cell differentiation (Figure 3D). DEG analysis followed by Kyoto Encyclopedia of Genes and Genomes pathway enrichment revealed abnormal activation of the TCR signalling pathway, Th17 cell differentiation and the MAPK signalling pathway in gMG (Figure 3E). Overall, these results indicate that CD4^+^ T cells in patients with gMG exhibit a more pro‐inflammatory phenotype, characterised by the upregulation of the Th17 pathway.

Similar to the analysis of CD4^+^ T‐cell sub‐compartments, six CD8^+^ T‐cell subsets were identified (Figure 3F). A naïve CD8^+^ population showed high expression of *SELL*, *LEF* and other naïve markers such as *CCR7* and *IL7R* (Figure 3G). Transitional CD8*^+^* T cells were characterised by elevated levels of *CD44* and *CD69*. Mucosal‐associated invariant T (MAIT) cells (*IL7R* and *CD69*) were identified using markers including *KLRB1/ KLRG1* and *ZBTB16*. Tem cells exhibited memory‐related molecules, such as *CD27*, *CD44* and *CXCR3*, whereas effector T (Teff) cells showed markers indicative of effector function and cytotoxicity, including *CX3CR1*, *FCGR3A*, *GZMB/GZMK*, *GNLY*, *PRF1* and *KLRB1/KLRD1/KLRG1* (Figure 3G). Compared to HCs, patients with gMG exhibited an expansion of transitional CD8*^+^* T cells and a reduction in MAIT cells (Figure 3H). The transcriptional profiles of CD8^+^ T cells also revealed *JUN*, *JUNB* and *EGR1* elevation in MG (Figure 3I). Additionally, levels of *SMAD1* and *SMAD3*, which are associated with tumour growth factor‐beta (TGF‐β) signalling, were higher in CD8^+^ T cells from patients with gMG. Overall, CD8^+^ T cells exhibit persistent chronic inflammation and impaired immune surveillance in patients with gMG.

### 3.4 Identification of altered B cells with differential responses to FcRn blockade in MG

**FIGURE 4:**
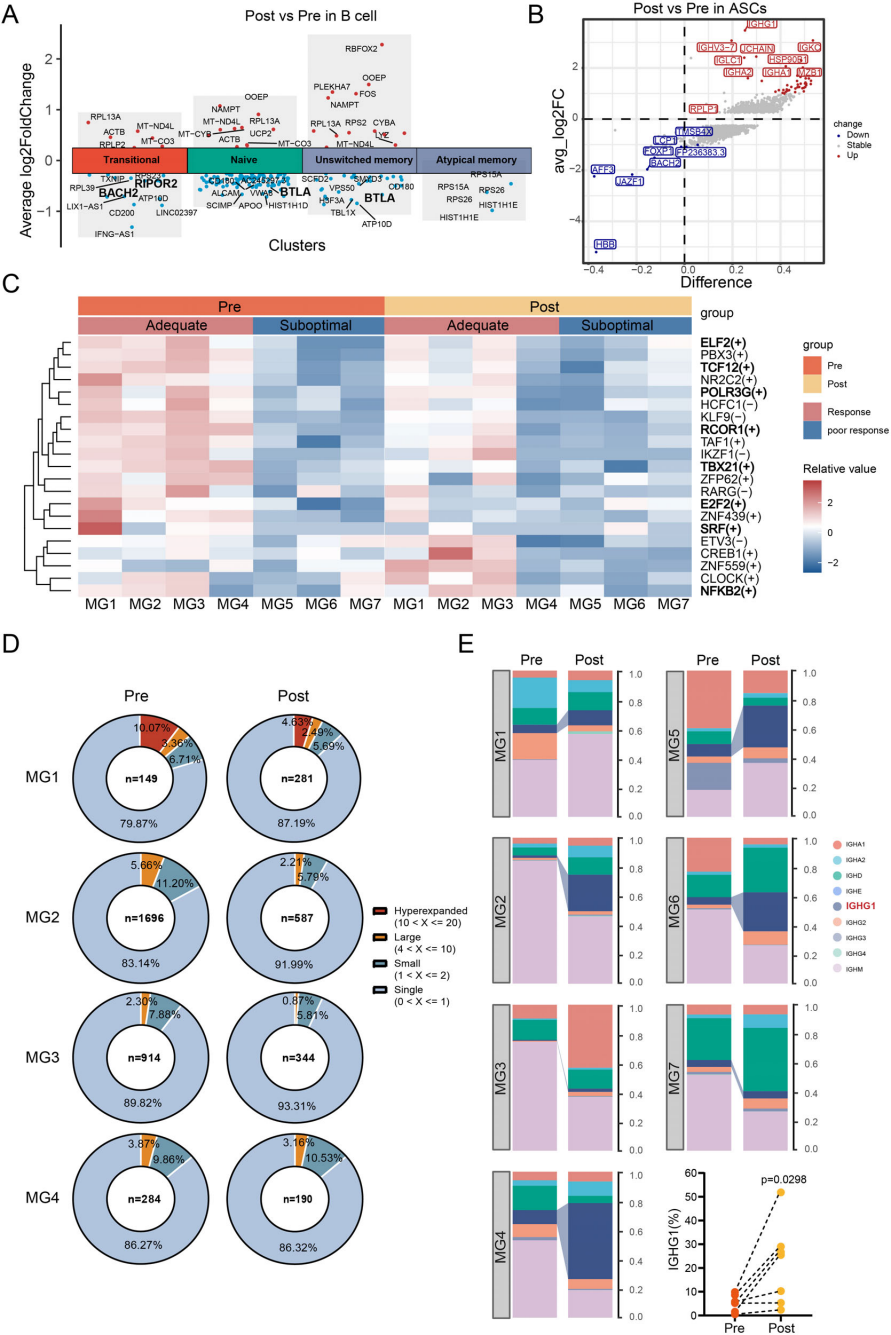
Gene expression, transcriptional regulon activity and BCR dynamics in B cells pre‐ and post‐neonatal Fc receptor (FcRn) blockade. (A) Differentially expressed genes (DEGs) in B‐cell subclusters from post‐treatment versus pre‐treatment samples. (B) Volcano plot of DEGs in antibody‐secreting cells (ASCs) post‐treatment. (C) Heatmap of transcription factor regulon activity in B cells from patients with adequate and suboptimal responses before and after FcRn blockade. The adequate and suboptimal responses were based on Myasthenia Gravis Activities of Daily Living (MG‐ADL) improvement (see Section 2). (D) Distribution of BCR clone sizes in four adequately responsive patients pre‐ and post‐treatment. (E) Distribution of BCR clone types across samples pre‐ and post‐treatment, highlighting *IGHG1* changes.

To investigate the impact of FcRn blockade on immune cell dynamics in patients with gMG, longitudinal profiling of B cells isolated before and after efgartigimod treatment was conducted (Figure S3A,B). Following the fourth infusion, the mean reduction in serum IgG levels exceeded 50%, indicating the overall effectiveness of FcRn blockade in the cohort (Table S3). Efgartigimod did not alter the relative proportions of B‐ and T‐cell subsets (Figures S3C and S4C). However, efgartigimod induced substantial transcriptional changes in B cells and ASCs (Figure 4A,B). In naïve and transitional B cells, transcripts such as *RIPOR2* and *BACH2* were significantly reduced (Figure 4A and Table S7). Loss of *BACH2* signifies differentiation towards ASCs.^14^ Additionally, *BTLA* was downregulated in naïve and unswitched memory B cells, which enhances T‐cell activation and proliferation by diminishing negative regulatory signals, thereby promoting stronger T–B cell interactions and potentially increasing antibody secretion.^15^ ASCs exhibited high transcriptional activity of immunoglobulin genes *IGHG1*, *IGHA1/IGHA2*, *IGHV3‐7*, *IGLC1/IGKC* and *JCHAIN*, with a notable reduction in *BACH2* (Figure 4B). Despite the substantial reduction in peripheral IgG levels after FcRn blockade, the differentiation potential of B cells into ASCs to express immunoglobulins remain prominent.

Among Cohort #2, four patients (MG 1–4) achieved an adequate response to efgartigimod. Analysis of transcription regulators revealed notable differences between patients with adequate and suboptimal responses. *TBX21* and *POLR3G* exhibited higher levels of activation in adequately responsive patients (Figure 4C). *POLR3G*, a gene involved in innate immunity and cell proliferation, senses non‐self dsDNA and activates type I interferon (IFN) and nuclear factor‐kappa B (NF‐κB) through the RIG‐I pathway.^16^ Similarly, *TBX21* regulates antiviral B‐cell responses and promotes IgG isotype switching. Conversely, patients with low expression of these TFs exhibited suboptimal responses to efgartigimod. In summary, the activation pattern of this TF cluster may indicate efgartigimod responsiveness.

Next, screening in a high‐resolution view of clonal relationships within B cells was performed by analysing transcriptomes and antigen receptor repertoires. Although most B‐cell clones were singletons, a small fraction of hyperexpanded or large clones was observed (Figures 4D and S3D). In the adequate‐response subgroup, these large clones shrank post‐treatment compared to pre‐treatment, indicating a reduction in clonal expansion. Furthermore, a comparison of IGH isotypes revealed an increase in *IGHG1* after treatment (Figure 4E), consistent with the transcriptional signatures of ASCs (Figure 4B). Following FcRn blockade, an increase in the clonal diversity of B cells and a higher number of class‐switched BCRs were observed, suggesting that a feedback mechanism was activated in response to IgG exhaustion. These results suggest potential biomarkers for treatment responsiveness and underscore the dynamic nature of B‐cell responses in patients with gMG following FcRn blockade.

### 3.5 FcRn blockade with efgartigimod inhibits Th17 cell activity

**FIGURE 5:**
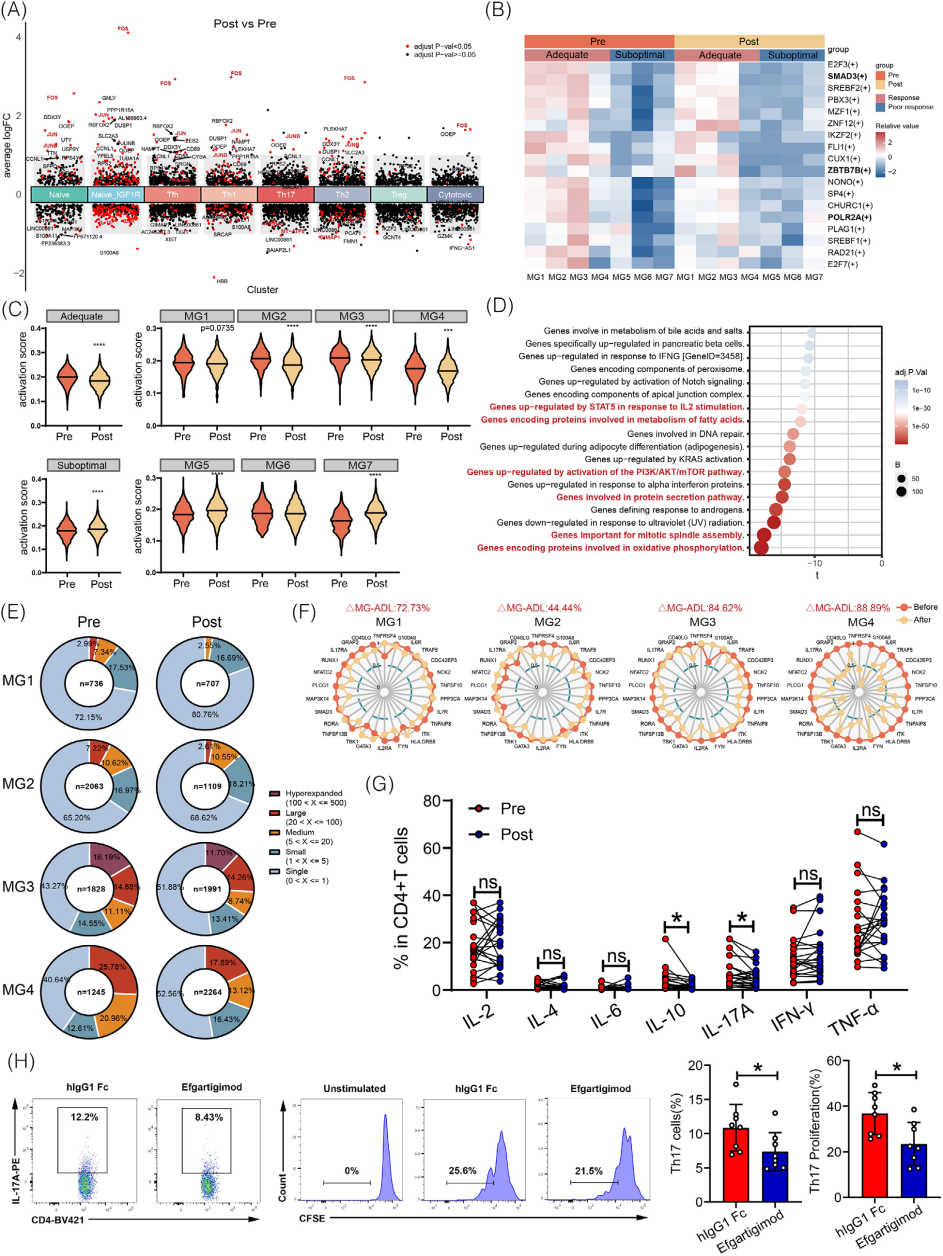
Gene expression, transcriptional regulon activity and TCR dynamics in CD4^+^ T cells pre‐ and post‐neonatal Fc receptor (FcRn) blockade. (A) Differentially expressed genes (DEGs) in CD4^+^ T‐cell subclusters from post‐treatment versus pre‐treatment samples. (B) Heatmap of transcription factor regulon activity in CD4^+^ T cells from adequate and suboptimal response groups before and after FcRn blockade. (C) Quantification of T‐cell activation scores in individual patients. Genes marking T‐cell activation are included in Table S2. (D) Gene set variation analysis of downregulated pathways in post‐treatment versus pre‐treatment groups. (E) Distribution of TCR clone sizes in four adequately responsive patients pre‐ and post‐treatment. (F) Radar plots of IL‐17 signalling pathway‐related gene expression in four adequately responsive patients. (G) Quantification of pro‐inflammatory and anti‐inflammatory cytokines in cohort #3 with FcRn blockade treatment (Table S3). (H) Flow cytometry validating the effects of efgartigimod on the differentiation and proliferation of Th17 cells. Notably, a significant reduction in Th17 cells and CFSE proliferation was determined in the dosing group (*p *< .05).

Given the significant expansion of CD4^+^ Tm cells in patients with gMG and their reduction following FcRn blockade, transcriptional changes in CD4^+^ T cells after efgartigimod treatment were examined. Genes in the *S100A* family, *GIMAP7*, and *IL7R* were broadly downregulated across various T‐cell subsets, including Th1, Th2, naïve T and Naïve_IGF1R cells, potentially impairing T‐cell survival and promoting apoptosis (Figure 5A and Table S8). A significant reduction in the transcription of *MT‐ATP8* and *AMBRA1* was observed, which may disrupt mitochondrial bioenergetics and autophagic flux in Th17 cells following treatment. Furthermore, the downregulation of *NLRC5* in Th2 cells, together with decreased *IL16* transcription in Naïve_IGF1R T cells, suggests a potential impairment in antigen presentation capacity upon FcRn blockade.

We identified notable differences in CD4^+^ cell transcriptional regulation between patients with adequate and suboptimal response. The downregulation of *E2F3* and *E2F7* transcriptional activity may limit T‐cell proliferation (Figure 5B). *ZBTB7B*, critical for T‐cell subset differentiation, particularly CD4^+^ T cells, may disrupt T‐cell subset balance and further suppress effector T‐cell function when its transcription is repressed. The reduced activity of *FLI1* and *SP4* may inhibit pro‐inflammatory cytokine expression, thereby attenuating T‐cell activation. In summary, the significant downregulation of these T‐cell‐associated TFs in patients with an adequate response to efgartigimod likely contributes to alleviating the pro‐inflammatory state of T cells in gMG by modulating their proliferation, differentiation and activation. Additionally, T‐cell activity is generally reduced in adequately responsive patients, whereas a slight increase is noted in patients with suboptimal response (Figure 5C). DEG analyses revealed dampened activity in pathways such as the IFNG response, IL‐2‐stimulated STAT5 activation and the PI3K/AKT/mTOR pathway following treatment (Figure 5D). Among the four patients with an adequate response, the clonal diversity of T cells increased after treatment, as evidenced by the contraction of large clones and the increased proportion of monoclonal populations (Figure 5E). Genes involved in IL‐17 signalling pathways, such as *IL17RA*, *TBK1*, *TRAF5* and *S100A8*, along with those related to Th17 cell differentiation, including *RUNX1*, *PLCG1*, *RORA*, *NFATC2*, *GATA3*, *PPP3CA* and *SMAD3*, exhibited reduced expression after FcRn blockade in these patients (Table S10). Notably, greater reduction of this gene cluster was linked to a higher rate of disease alleviation (△MG‐ADL) (Figures 5F and S4E and Table S9). Flow cytometry validated the downregulation of IL‐17A in CD4^+^ T cells post‐treatment (Figure 5G). In vitro, efgartigimod significantly decreased Th17 cell differentiation and CFSE‐detected proliferation in the dosing group (Figure 5H). Collectively, these findings indicate that genes associated with T‐cell survival and activation pathways were downregulated in CD4^+^ T cells in gMG patients after FcRn blockade, with the IL‐17A signalling pathway being the most significantly inhibited.

### 3.6 Cell interactions are active in MG and antigen presentation is reduced after FcRn blockade

**FIGURE 6:**
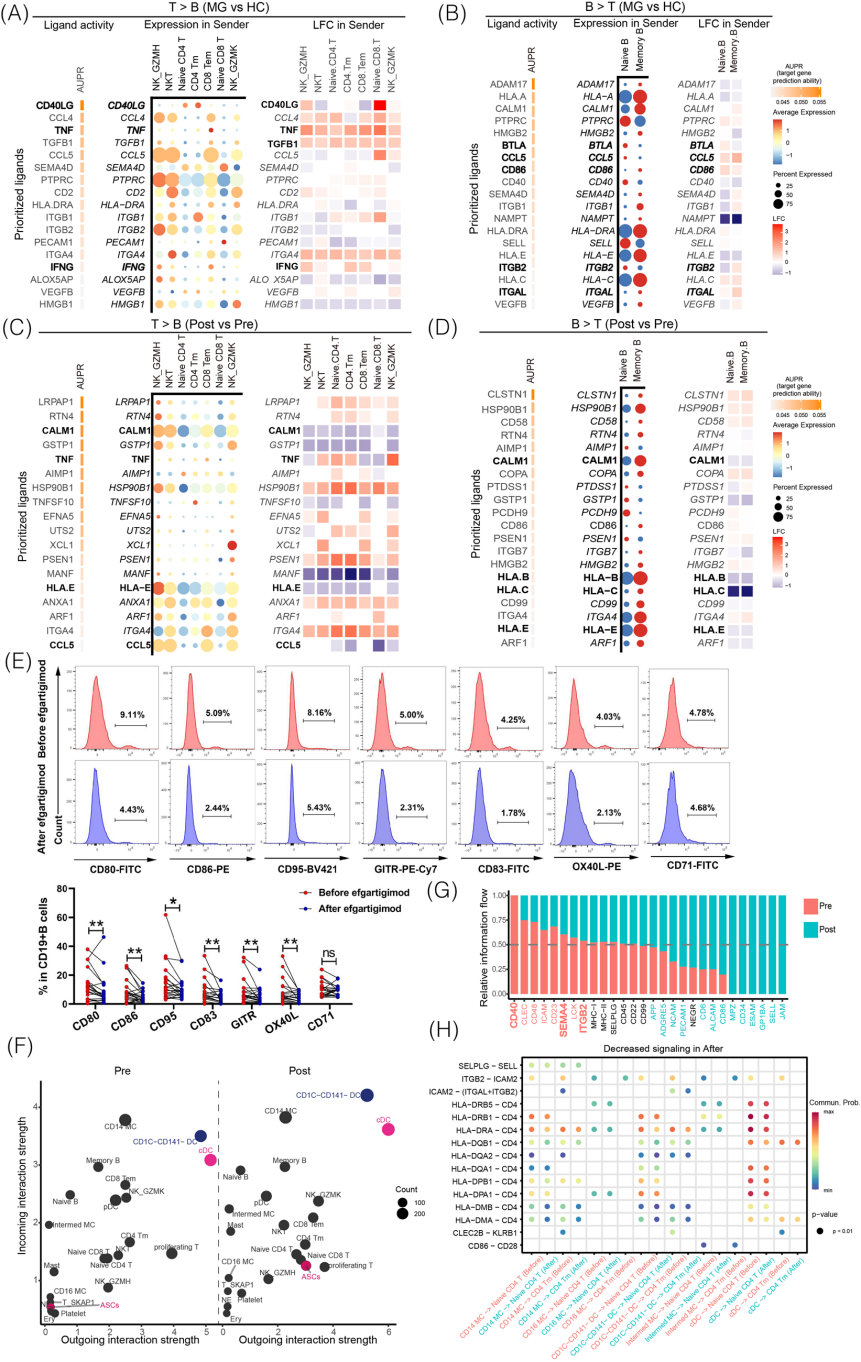
Intercellular communication and antigen presentation in neonatal Fc receptor (FcRn) blockade. NicheNet analysis of intercellular signals from T/NK cells to B cells (A) and signals from B cells to T/NK cells (B) in healthy control (HC) and generalised myasthenia gravis (gMG) groups (ranked by ligand activity). Similar analyses were performed for the comparison of signalling pre‐ and post‐FcRn blockade (C and D). (E) Flow cytometry analysis showing reduced expression of antigen‐presenting markers and T‐cell co‐stimulators in CD19^+^ B cells post‐treatment. (F) Scatter plots of incoming and outgoing signalling strengths in paired peripheral blood mononuclear cell (PBMC) samples pre‐ and post‐treatment. (G) Bar plots showing proportions of overall signalling flows in PBMC samples pre‐ and post‐treatment. (H) Dot plots highlighting significant changes in cell‐to‐cell communication pathways involving monocytes, dendritic cells and CD4^+^ T cells pre‐ and post‐treatment.

T–B cell crosstalk in patients with gMG was investigated. Using NicheNet, the analysis revealed elevated interactions between T and NK cells towards B cells in patients with gMG compared to HCs (Figure 6A). Notably, significant IFNG–IFNGR1/2 interactions were detected in T–B cell (Figure S5A). *IFNG* enhances peptide generation by inducing lysosomal proteolytic cleavage preferences, playing a role in antigen presentation pathways.^17^, ^18^ Increased *IFNG* transcription may drive atypical memory B‐cell production.^19^ Additionally, TNF and *TGFB1* signals were broadly enhanced in T/NK cells of MG (Figure 6A). A significant *CD40LG* flow was detected from naïve CD8^+^ T cells (Figure 6A). Previous studies have shown that CD8^+^ T cells can stimulate B‐cell growth and differentiation via CD40L–CD40 interactions.^20^ Both *ITGAL* and *ITGB2* signal flows from memory B cells were significant in MG (Figure 6B). *CCL5* was also elevated in naïve and memory B cells of MG. Additionally, CD86 expression on B cells, binding CD28 on T cells, was elevated in naïve and memory B cells (Figure S5B). Taken together, the enhanced signal flows between T/NK cells and B cells underscore the elevated cell surface‐mediated signalling and provide a supportive niche for T–B cell interactions.

We then examined the effects of FcRn blockade on the interactions between T/NK and B cells (Figure 6C,D). Specifically, *TNF* signalling from different NK and T‐cell subsets to B cells showed varied patterns, with elevation from CD4^+^ Tm cells but reduction from CD8^+^ Tm cells. *CCL5* signalling from CD4^+^ Tm cells and naïve CD8^+^ T cells were significantly reduced after FcRn blockade (Figure 6C), as were flows of *HLA‐B*, *HLA‐C* and *HLA‐E* in memory B cells (Figure 6D). Additionally, interactions including *CD40LG*–*CD40*, *ADGRE5*–*CD55* and *SELPLG*–*SELL* were significantly reduced, particularly in naïve CD4^+^ T and Tm cell interactions with naïve and memory B cells (Figure S5C). Flow cytometry revealed downregulated antigen‐presenting markers (CD80, CD86, CD95 and CD83) and T‐cell co‐stimulators (GITR and OX‐40L) on CD19^+^ B cells post‐treatment (Figure 6E).

DCs, monocytes, and CD4^+^ T‐cell subsets showed significant reductions in interaction strength after FcRn blockade (Figure 6F), consistent with the substantial DEGs observed in these subgroups (Figure 1C). Supplementary analyses further revealed that monocytes in gMG exhibited pronounced pro‐inflammatory signatures and actively engaged in ligand–receptor interactions that were attenuated following treatment (Figure S6). The frequency of IL‐15⁺ monocytes was elevated in gMG patients compared to HCs, and declined following FcRn blockade in adequately responsive patients (Figure S6A). DEGs revealed pronounced upregulation of functional categories in monocytes from gMG patients relative to HCs. These included chemokines (*CCL3*, *CCL4*, *CCL5*, *CXCL2*, *CXCL3*), pro‐inflammatory mediators (*IL1B*), antigen presentation and T‐cell activation molecules (*HLA‐DQB1*, *CD83*) and key transcriptional regulators of inflammatory differentiation (*EGR1*, *KLF4*) (Figure S6B). Of particular interest, *CCL5* expression was significantly reduced in both CD14⁺ and CD16⁺ monocytes following efgartigimod treatment (Figure S6C). Intercellular communication analyses further demonstrated enhanced ligand–receptor interactions involving CCL3/4/5–CCR5 in gMG patients compared to HCs (Figure S6D,E; left). These pro‐inflammatory circuits, particularly T/NK–monocyte/DC interactions involving CCL3/4/5–CCR1 and CD40LG–ITGA5/CD40, were markedly attenuated by FcRn blockade (Figure S6D,E; right). GSVA demonstrated downregulation of complement signalling, CD22‐mediated BCR regulation, chemokine receptor interactions, and integrin‐ligand binding following treatment (Figure S6F). Pseudotime trajectory analysis showed a reduction in the proportion of CD16⁺ monocytes occupying terminal pro‐inflammatory states after treatment, suggesting that FcRn blockade may inhibit the terminal differentiation of inflammatory monocyte subsets (Figure S6G).

Converse to myeloid cells, being strong sources of bidirectional signals, ASCs displayed low interaction activity in MG; however, interaction strength increased after FcRn blockade (Figure 6F). This finding was consistent with previous observations of high transcriptional activity of immunoglobulin genes (*IGHG1*, *IGHA1/2*, *IGHV3‐7*, *IGLC1/IGKC* and *JCHAIN*) in ASCs post‐treatment (Figure 4B). Overall, *CD40*, *ICAM* and *ITGB2* signalling flows were significantly reduced post‐treatment (Figure 6G). Focusing on interactions between myeloid cells and CD4^+^ T cells, a significant decrease in antigen presentation, primarily via MHC II, was observed (Figure 6H). Antigen presentation‐related pairs between MCs/DCs and naïve or memory CD4^+^ T cells were significantly reduced after FcRn blockade, supporting the immune‐modulatory mechanisms of FcRn blockade. MHC class II receptor–CD4 interactions were reduced, underscoring the downregulation of antigen presentation after FcRn blockade. In other analysed pairs, ITGB2–ICAM2 interactions were elevated in most myeloid cells and CD4^+^ T‐cell pairs of patients with gMG, but were absent after FcRn blockade (Figure 6H). These results suggest that FcRn blockade dampened antigen presentation processes but enhanced immunoglobulin transcription in MG.

## 4 DISCUSSION

The pathogenesis of gMG is thought to be mediated by aberrant B and T cells. Nevertheless, the immune landscape and molecular signatures of these cells in gMG remain poorly understood. In this study, we demonstrated the highly active property of B cells in the peripheral blood of patients with gMG. A significant expansion of atypical B cells was observed, indicative of active somatic hypermutation and antigen‐driven activation.

Although FcRn‐targeting therapy is widely used to treat gMG, little is known about its effects beyond reducing serum IgG and AChR levels, particularly its molecular impact on peripheral immune cells. In this study, we aimed to provide an unbiased roadmap of the peripheral immune compartment and the response to FcRn blockade in gMG. Since FcRn blockade with efgartigimod does not directly alter B‐cell subset distribution, its effectiveness in gMG appears influenced by specific transcriptional factors. Importantly, the initial upregulation of *TBX21* in B cells may serve as a key marker for predicting a favourable response to efgartigimod treatment. T‐bet^+^ B cells represent a specific pathogenic subset that is frequently expanded in autoimmune diseases such as systemic lupus erythematosus and rheumatoid arthritis.^21^, ^22^ These cells may drive disease progression through the secretion of high levels of autoantibodies or as antigen‐presenting cells promoting inflammatory Th responses.^23^, ^24^ In this study, the abnormal expansion of T‐bet^+^ B cells in MG could not be reversed by FcRn blockade. However, *TBX21* activation was higher in patients who responded favourably to efgartigimod, with more pronounced downregulation post‐treatment. This suggests that efgartigimod impacts transcriptional activity rather than the number of T‐bet^+^ B cells. Within the T‐bet^+^ B‐cell subset, T‐bet expression exhibits heterogeneity, correlating with phenotypic differences in B cells.^23^ T‐bet expression, heavily dependent on IFN‐γ signalling, enhances antibody production and predicts the long‐term characteristics of B‐cell subsets and their antibody responses.^25^, ^26^ In summary, our findings highlight the potential of transcriptional activity, particularly *TBX21* activation, as a biomarker for the efficacy of efgartigimod in treating MG, attributed to the central role of T‐bet^+^ B cells in pathogenic IgG production and antigen presentation.

An increase in *IGHG* transcription was observed, particularly *IGHG1* after efgartigimod treatment. ASCs exhibited lowered transcriptional activity of *BACH2* following FcRn blockade, which may contribute to the upregulation of immunoglobulin gene transcription. Moreover, the increased strength of both incoming and outgoing interactions in ASCs suggests enhanced communication within the immune microenvironment. This may reflect a compensatory immune response, whereby ASCs intensify their interactions with other immune cells, potentially to sustain autoantibody production or maintain immune homeostasis despite the reduction in IgG levels. In addition, the downregulation of *TIGIT* in Tfh cells, observed after treatment, may stimulate B‐cell proliferation and antibody responses (Figure 5A and Table S8). *FOS*, *JUN* and *JUNB* were broadly upregulated across multiple CD4^+^ T‐cell subsets, indicating persistent T‐cell activation and impaired immune regulation despite FcRn blockade.^27^ This underscores the incomplete suppression of immune activity by FcRn blockade alone and may imply the necessity for adjunctive therapies to further inhibit B‐cell activity and autoantibody production. Future clinical trials are needed to explore the combination of FcRn blockade with upstream‐targeting strategies to enhance and prolong therapeutic efficacy.

These findings revealed abnormal activation of the TCR signalling pathway and Th17 cell differentiation in MG. Following FcRn blockade, there was a notable downregulation of Th17 cell activation and IL‐17A expression, which raises questions regarding the mechanisms by which FcRn blockade may impair Th17 activation. First, FcRn blockade attenuated signalling pathways that promote Th17 differentiation. Genes associated with IL‐17 signalling pathways, such as *IL17RA*, *TBK1*, *TRAF5* and *S100A8*, as well as those involved in Th17 differentiation, including *RUNX1*, *PLCG1*, *RORA*, *NFATC2*, *GATA3*, *PPP3CA* and *SMAD3*, were downregulated after FcRn blockade, particularly in adequately responsive patients. Second, the antigen presentation‐related interactions between MCs/DCs and naïve or memory CD4^+^ T cells were significantly reduced post‐FcRn blockade, especially for MHC class II receptor–*CD4* interactions. This finding is consistent with previous research demonstrating that FcRn plays a critical role in the transport and retention of IgG and immune complexes, which influence antigen presentation.^28^, ^29^ *ITGB2*–*ICAM2* interactions were elevated in most myeloid cells and CD4^+^ T‐cell pairs of patients with gMG, but were absent after FcRn blockade. FcRn blockade could disrupt the stability or strength of these synapses, influencing the transfer of signals necessary for Th17 differentiation. In this regard, FcRn blockade could impair antigen presentation and immunological synapse formation of Th17 cells, thereby potentially impairing their activation and subsequent IL‐17A production. In addition, the downregulation of *MT‐ATP8* and *AMBRA1* in Th17 cells post‐treatment may indicate alterations in mitochondrial bioenergetics and autophagic flux, affecting Th17 cell viability, activation and differentiation. Mitochondrial oxidative phosphorylation is essential for sustaining cytokine production, particularly IL‐17, and maintaining cellular proliferation. In summary, FcRn blockade likely impacts the activation of Th17 cells and their IL‐17A expression by diminishing IL‐17 signalling pathways, altering antigen presentation and immunological synapse formation, and interfering with Th17 cellular energy balance. Further studies are needed to elucidate these intricate pathways and their implications for Th17 immunity.

Given the heterogeneity of MG, this study used longitudinal analyses integrating immune repertoire sequencing to identify the underlying mechanisms of disease pathogenesis and its diverse treatment responses. This approach aids in understanding the differential treatment outcomes and promotes the development of personalised therapeutic strategies.^30^, ^31^, ^32^ However, some limitations should be noted. First, the patients received only one cycle of efgartigimod treatment, and the effects of multiple cycles of prolonged FcRn blockade on the peripheral blood immune microenvironment remain unclear. Second, the relatively small sample size limits statistical power. Further validation of bioinformatic results is needed in larger external cohorts. Additionally, future research should incorporate multi‐omics integrative analyses with cross‐validation to achieve a more comprehensive understanding of the underlying mechanisms. Notably, given that the myeloid lineage exhibited the highest number of DEGs after FcRn blockade, further investigations focusing on the myeloid compartment hold significant promise for uncovering novel insights into the pathophysiology and treatment response in anti‐AChRab‐positive gMG. Furthermore, our findings suggest that FcRn blockade, while effective, does not fully suppress the pathological processes of gMG or completely address clinical needs. Targeting upstream immune components is also critical to enhance and prolong therapeutic efficacy.

## 5 CONCLUSIONS

This study identified the molecular features in patients with gMG. These findings suggest that mature B cells, including atypical B cells, are highly activated, and the interplay between B cells and other lymphocytes plays a significant role in gMG. Patients with gMG show varying treatment responses to FcRn blockade. Importantly, increased class‐switched BCRs following FcRn blockade provide valuable insights into potential therapeutic regimens in gMG. The negative feedback mechanisms driven by IgG exhaustion allow activated B cells to retain their potential for pathogenic antibody secretion. Therefore, targeting upstream immune components is essential for achieving prolonged therapeutic efficacy.

## AUTHOR CONTRIBUTIONS

*Conceptualisation*: Chao Zhang and Zhigang Cai. *Resources*: Hui‐Ning Li, Xiao‐Yu Huang and Bo Zhang. *Investigation*: Xiao‐Yu Huang, Lijie Zhu and Zhirui Liu. *Formal analysis and data curation*: Jingjing Liu and Zhigang Cai. *Visualisation*: Hui‐Ning Li, Jingjing Liu and Xiao‐Yu Huang. *Validation*: Hui‐Ning Li, Jingjing Liu and Xiao‐Yu Huang. *Supervision*: Chun‐Sheng Yang and Shixiong Huang. *Writing—original draft*: Hui‐Ning Li and Jingjing Liu. *Writing—review and editing*: Fu‐Dong Shi, Chao Zhang and Zhigang Cai.

## CONFLICT OF INTEREST STATEMENT

The authors declare they have no conflicts of interest.

## ETHICS STATEMENT

The studies were conducted in compliance with local legislation and institutional requirements. The ethics committees and institutional review boards of Tianjin Medical University General Hospital and Hainan General Hospital approved the study protocol and sampling. This study adhered to the principles set forth in the Declaration of Helsinki. Written informed consent was obtained from all participants.

## Supporting information



Supporting Information

## Data Availability

Single‐cell RNA‐seq data have been deposited at Genome Sequence Archive (GSA in BIG Data Center, Beijing Institute of Genomics, Chinese Academy of Sciences) and are publicly available  upon reasonable request. Anonymised data will be shared on request from any qualified investigators.
