# Supplementary material for: Single‐cell atlas reveals heterogeneous response to FcRn blockade in anti‐AChR antibody‐positive generalised myasthenia gravis
Source: Clin Transl Med. 2025 Aug 6;15(8):e70436. doi: 10.1002/ctm2.70436 (PMC12328247; doi:10.1002/ctm2.70436)
Supplement: Supplementary file 1 — Supporting Information [file CTM2-15-e70436-s001.docx]

**Supplementary online Materials**

**Table of Contents**

[Supplementary Tables 1](#_Toc2046562701)

[Supplementary Figures and Figure Legends 4](#_Toc333741708)

[Supplementary Figure 1 4](#_Toc2117084039)

[Supplementary Figure 2 5](#_Toc174896330)

[Supplementary Figure 3 6](#_Toc1724989214)

[Supplementary Figure 4 7](#_Toc864485198)

[Supplementary Figure 5 8](#_Toc1675850831)

[Supplementary Figure 6 9](#_Toc1776886212)

**Supplementary Tables**

**Supplementary Table 1. Key resources table**

| REAGENT OR RESOURCE | SOURCE | IDENTIFIER |
| --- | --- | --- |
| Antibodies | | |
| Anti-human CD3 | BD Biosciences | Cat#: 566783; RRID: AB_2869864 |
| Anti-human CD4 | BD Biosciences | Cat#: 562424; RRID: AB_11154417 |
| Anti-human IL-2 | Biolegend | Cat#: 500307; RRID: AB_315093 |
| Anti-human IL-4 | Biolegend | Cat#: 500824; RRID: AB_2126747 |
| Anti-human IL-6 | Biolegend | Cat#: 501120; RRID: AB_2572042 |
| Anti-human IL-10 | Biolegend | Cat#: 501418; RRID: AB_2561286 |
| Anti-human IL-17A | Biolegend | Cat#: 512306; RRID: AB_961394 |
| Anti-human IFN-γ | Biolegend | Cat#: 506528; RRID: AB_2566186 |
| Anti-human TNF-α | Biolegend | Cat#: 502944; RRID: AB_2562869 |
| Anti-human IL-17A | Biolegend | Cat#: 512315; RRID: AB_2295923 |
| Anti-human CD19 | Biolegend | Cat#: 302212; RRID: AB_314241 |
| Anti-human CD80 | Biolegend | Cat#: 375406; RRID: AB_2890818 |
| Anti-human CD86 | Biolegend | Cat#: 374206; RRID: AB_2721632 |
| Anti-human CD95 | Biolegend | Cat#: 305624; RRID: AB_10895755 |
| Anti-human GITR | Biolegend | Cat#: 371224; RRID: AB_2687170 |
| Anti-human CD83 | Biolegend | Cat#: 305306; RRID: AB_314513 |
| Anti-human OX40L | Biolegend | Cat#: 326308; RRID: AB_2207272 |
| Anti-human CD71 | Biolegend | Cat#: 334104; RRID: AB_2201482 |
| Biological samples |  |  |
| Fresh PBMC samples | Tianjin Medical University General Hospital | N/A |
| Fresh PBMC samples | Hainan General Hospital | N/A |
| Cryopreserved PBMC samples | Tianjin Medical University General Hospital | N/A |
| Recombinant proteins | | |
| Recombinant human CD3 monoclonal antibody | eBioscience | Cat#:16-0037-38; RRID: AB_468854 |
| Recombinant human CD28 monoclonal antibody | eBioscience | Cat#:16-0289-81; RRID: AB_468926 |
| Critical commercial assays | | |
| CFSE Cell Division Tracker Kit | Biolegend | Cat#:423801 |
| eBioscience™ Intracellular Fixation & Permeabilization Buffer Set | Invitrogen | Cat#:88-8824-00 |
| Deposited data | | |
| Single-cell RNA sequencing data | This paper | https://ngdc.cncb.ac.cn/gsub/ |
| Software and algorithms | | |
| R (v3.5.1, v4.3.0) | R Development Core Team | https://cran.r-project.org |
| Seeksoultools (v1.2.0) | SeekGene BioSciences | http://seeksoul.seekgene.com |
| Seurat (v4.4.0) | Satija Lab | https://satijalab.org/seurat |
| Harmony (v1.0) | Korsunsky et al.[1] | https://github.com/immunogenomics/harmony |
| NicheNet (v1.1.1) | Browaeys et al.[2] | https://github.com/saeyslab/nichenetr |
| GraphPad Prism version 10.0 | GraphPad software | https://www.graphpad.com/ |
| GSVA (v1.44.5) | Hänzelmann et al.[3] | https://github.com/rcastelo/GSVA |
| Monocle2 (v2.26.0) | Trapnell et al.[4] | https://cole-trapnell-lab.github.io/monocle-release |
| pySCENIC (v0.12.1) | Sande et al.[5] | https://github.com/aertslab/pySCENIC |
| scRepertoire (v1.8.0) | Borcherding et al.[6] | https://github.com/ncborcherding/scRepertoire |
| CellChat (v1.6.1) | Jin et al.[7] | https://github.com/sqjin/CellChat |
| FlowJo | BDBiosciences | https://flowjo.com/ |
| Other | | |
| Ficoll gradient separation | TBD Sciences | Cat#:LTS1077 |
| eBioscience™ Cell Stimulation Cocktail | Invitrogen | Cat#:00-4970-93 |
| Efgartigimod | Vetter Pharma-Fertigung GmbH & Co. KG | N/A |

**Supplementary Table 2. Genes marking T-cell activation**

**Supplementary Table 3. Demographic information of patients with MG enrolled for single-cell RNA sequencing and vitro validation.**

|  | **Cohort 1 (n = 10)** | **Cohort 2 (n = 7)** | **Cohort 3 (n = 21)** |
| --- | --- | --- | --- |
| **Age, years, mean (SD)** | 60.0 ± 11.9 | 60.3 ± 15.2 | 55.10±3.99 |
| **Female, No. (%)** | 6 (60) | 2 (29) | 14 (66.67) |
| **Age of onset, No. (%)** |  |  |  |
| **EOMG (age＜50 y)** | 2 (20) | 1 (14) | 7 (33) |
| **LOMG (age≥50 y)** | 8 (80) | 6 (86) | 14 (67) |
| **Disease duration, years, median^⁎^** | 3.9 (0.2, 9.1) | 8.0 (0.2, 9.0) | 1.0 (0.2, 5) |
| **Previous thymectomy, No. (%)** | 1 (10) | 3 (43) | 5 (24) |
| **MG-QMG, median^⁎^** | 22 (13, 26) | 20 (14, 23) | 9 (8,16) |
| **MG-ADL, median^⁎^** | 9 (4, 13) | 9 (8, 13) | 6 (5,11) |
| **MGFA classification** |  | |  |
| II, No. (%) | 4 (40) | 3 (43) | 12 (57.14) |
| III, No. (%) | 2 (20) | 3 (43) | 8 (38.10) |
| IV, No. (%) | 4 (40) | 1 (14) | 1 (4.76) |
| **AChR-ab levels (nmol/L), mean (SD)** | / | 12.4 ± 4.0 → 7.7 ± 4.2 | / |
| **Total IgG levels (g/L), mean (SD)** | 9.4 ± 3.2 10.7 ± 3.7 → 5.0 ± 3.5 12.02 ± 5.58 → 4.96 ± 1.55 | | |

**^⁎^**Scales are shown as median (quartile). MG, myasthenia gravis. QMG, Quantitative Myasthenia Gravis; ADL, activities of daily living; MGFA, Myasthenia Gravis Foundation of America. Total IgG levels were measured using a turbidimetric inhibition immunoassay, and AChR antibody levels were assessed by radioimmunoassay.

**Supplementary Table 4. Differentially expressed genes in blood B cells from patients with MG versus healthy controls**

**Supplementary Table 5. Differentially expressed genes across each blood B-cell subset from patients with MG versus healthy controls**

**Supplementary Table 6. Differentially expressed transcriptional factors in ASCs from patients with MG versus healthy controls**

**Supplementary Table 7. Differentially expressed genes across each blood B-cell subset after FcRn blockade**

**Supplementary Table 8. Differentially expressed genes across each blood CD4^+^ T-cell subset after FcRn blockade**

**Supplementary Table 9. Downregulated genes in blood CD4^+^ T cells from patients with MG with adequate response to FcRn blockade**

[Supplementary Table 2, 4–9 are provided as separate Excel spreadsheets.](https://pan.baidu.com/s/1N6toh3djVmXyzjHhsrVmTg)

**Supplementary Table 10. DEGs in CD4^+^ T cells related to IL-17A**

| **IL-17 signaling pathway** | | **Th17 cell differentiation** | | |
| --- | --- | --- | --- | --- |
| FOS | JUN | FOS | JUN | SMAD2 |
| **S100A8** | NFKBIA | **RUNX1** | **SMAD3** | STAT3 |
| HSP90AB1 | **TRAF5** | NFKBIA | **PPP3CA** | STAT5A |
| **IL17RA** | CASP8 | IL6R | HSP90AB1 | TGFBR1 |
| IKBKB | HSP90AA1 | IL2RA | **RORA** | PPP3R1 |
| TRADD | TNFAIP3 | **PLCG1** | **NFATC2** | MAPK9 |
| TAB2 | MAP3K7 | NFATC3 | **GATA3** | TGFBR2 |
| TRAF3 | **TBK1** | PPP3CC | IKBKB | NFKB1 |
| MAPK14 | USP25 | STAT5B | CD3D | HLA-DRB1 |
| IKBKE | CASP3 | HSP90AA1 | IL6ST | NFATC1 |
| MAPK9 | NFKB1 | STAT6 | IL2RG | MAPK1 |
| ELAVL1 | MAPK1 | HLA-DRB5 | PRKCQ | TGFB1 |
| JUND | MAPK6 | PPP3CB | HIF1A | IFNGR1 |
| MAPK8 | TNF | MAPK14 | MTOR | MAPK8 |
| RELA |  | HLA-DRA | STAT1 | LAT |
|  |  | CD4 | RELA | IL27RA |

**References**

1. Korsunsky I, Millard N, Fan J, et al (2019) Fast, sensitive and accurate integration of single-cell data with Harmony. Nat Methods 16:1289–1296. <https://doi.org/10.1038/s41592-019-0619-0>

2. Browaeys R, Saelens W, Saeys Y (2020) NicheNet: modeling intercellular communication by linking ligands to target genes. Nat Methods 17:159–162. <https://doi.org/10.1038/s41592-019-0667-5>

3. Hänzelmann S, Castelo R, Guinney J (2013) GSVA: gene set variation analysis for microarray and RNA-Seq data. BMC Bioinform 14:7. <https://doi.org/10.1186/1471-2105-14-7>

4. Trapnell C, Cacchiarelli D, Grimsby J, et al (2014) The dynamics and regulators of cell fate decisions are revealed by pseudotemporal ordering of single cells. Nat Biotechnol 32:381–386. <https://doi.org/10.1038/nbt.2859>

5. Sande BV de, Flerin C, Davie K, et al (2020) A scalable SCENIC workflow for single-cell gene regulatory network analysis. Nat Protoc 15:2247–2276. <https://doi.org/10.1038/s41596-020-0336-2>

6. Borcherding N, Bormann NL, Kraus G (2020) scRepertoire: An R-based toolkit for single-cell immune receptor analysis. F1000Research 9:47. <https://doi.org/10.12688/f1000research.22139.2>

7. Jin S, Guerrero-Juarez CF, Zhang L, et al (2021) Inference and analysis of cell-cell communication using CellChat. Nat Commun 12:1088. <https://doi.org/10.1038/s41467-021-21246-9>

**Supplementary Figures and Figure Legends**

**Supplementary Figure 1**

**
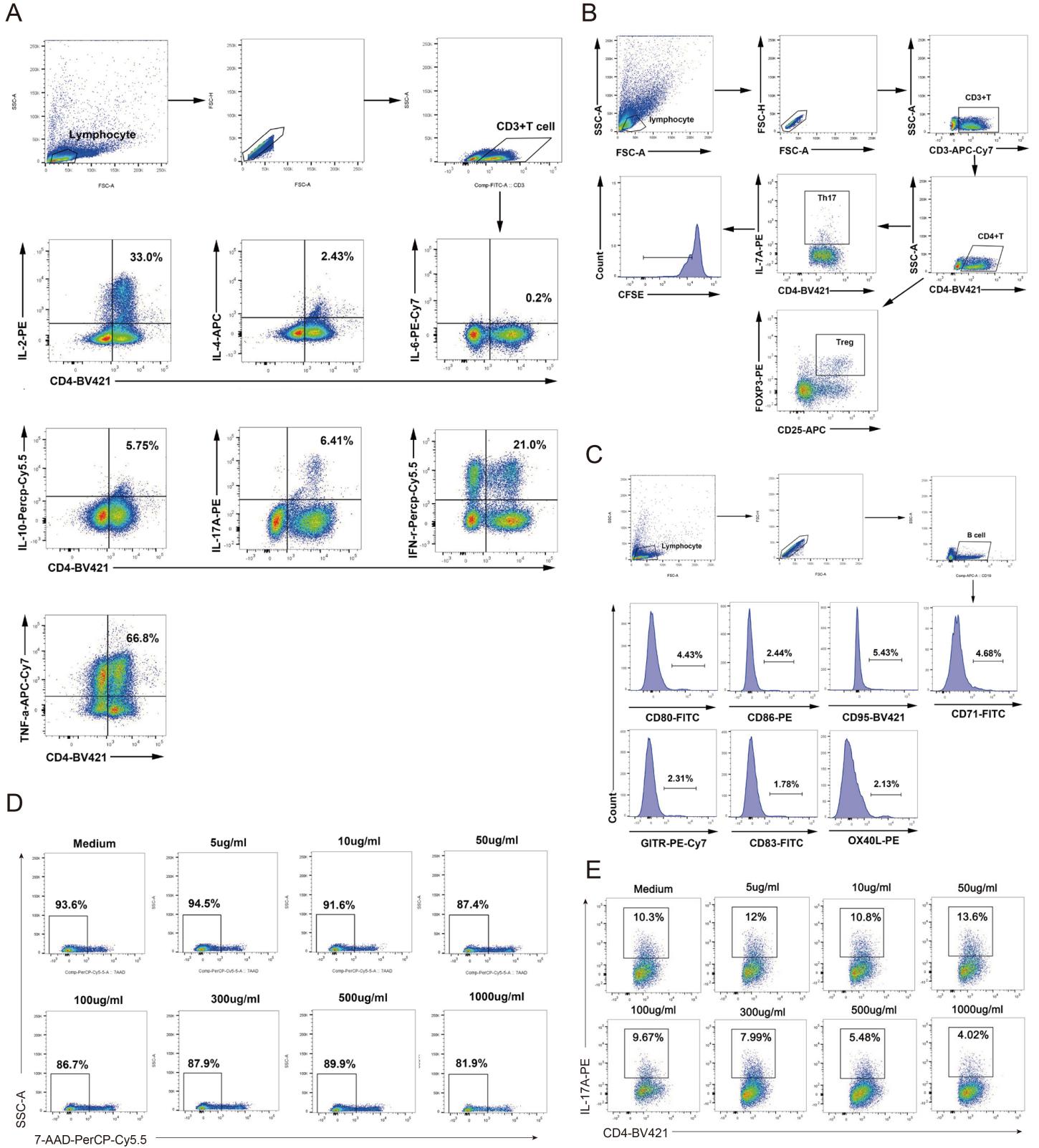
**

1. Flow cytometry gating strategy to detect intracellular cytokine in CD4^+^T cell before and after efgartigimod treatment.
2. Flow cytometry gating strategy to detect Th17 cell expression and proliferation, and Treg cell *in Vitro*.
3. Flow cytometry gating strategy to detect B-cell antigen presentation marker.
4. The dose-effect diagram of different doses of efgartigimod on PBMCs.
5. The dose-effect diagram of different doses of efgartigimod on the expression of Th17 cells.

# **Supplementary Figure 2**

**
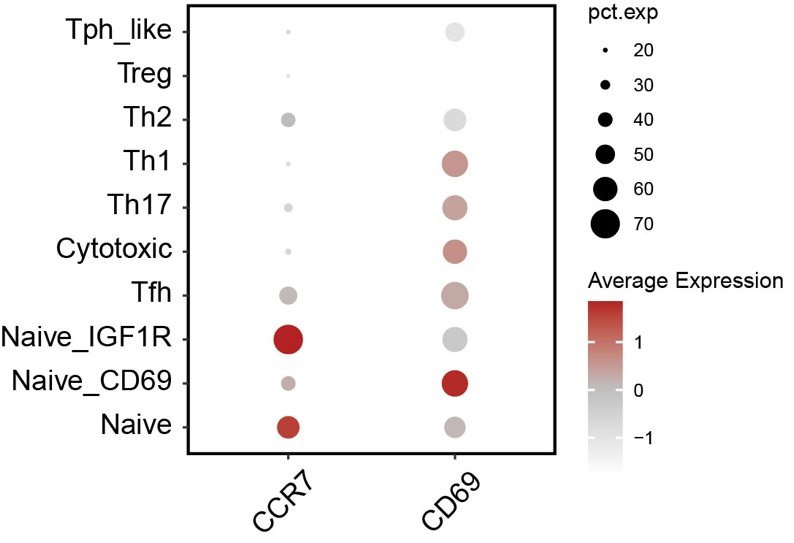
**

**Bubble heatmap illustrating the relative expression levels of stemness markers (CCR7 and CD62) across T-cell subclusters.**

**Supplementary Figure 3**

**
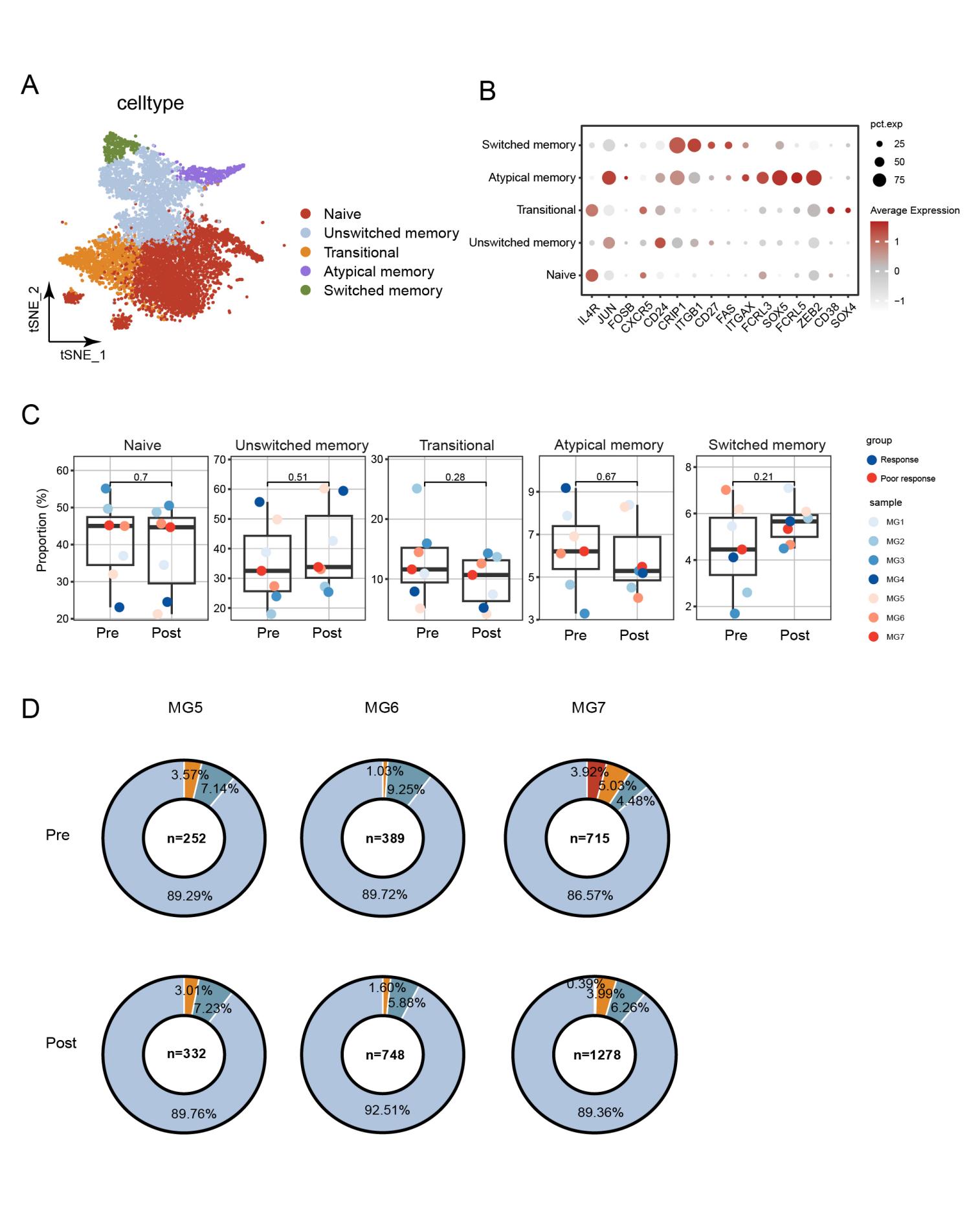
**

1. UMAP plot displaying ten B-cell subtypes in paired PBMC samples from seven patients with MG, pre- and post-FcRn blockade treatment.
2. Relative expression of indicated marker genes across B-cell subtypes.
3. Boxplots showing the proportions of B-cell subclusters pre- and post-FcRn blockade treatment. P values are marked.
4. Distributions of BCR clone size in the three gMG individuals with suboptimal response. The distributions are shown for the samples from pre- and post-treatment.

**Supplementary Figure 4**

**
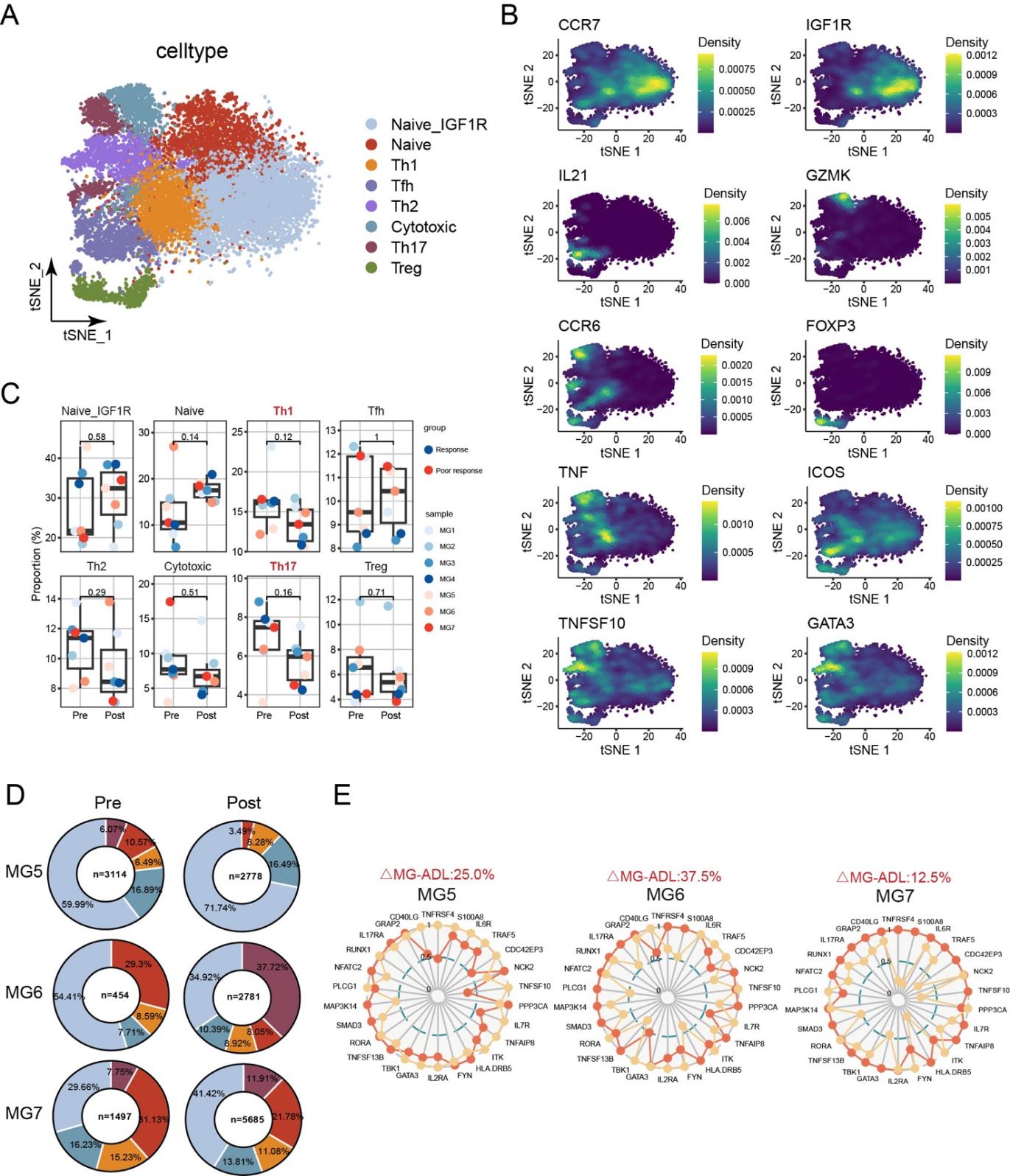
**

1. UMAP plot displaying ten CD4^+^ T-cell subtypes in paired PBMC samples from seven patients with MG, pre- and post-FcRn blockade treatment.
2. Relative expression of indicated marker genes across CD4^+^ T-cell subtypes.
3. Boxplots showing the proportions of CD4^+^ T subclusters pre- and post-FcRn blockade treatment. P values are marked.
4. Distributions of TCR clone size in the three gMG individuals with suboptimal response. The distributions are shown for the samples from pre- and post-treatment.
5. Radar plots showing expression of IL-17 signaling pathway-related genes in samples in the three gMG individuals with suboptimal response.

**Supplementary Figure 5**

**
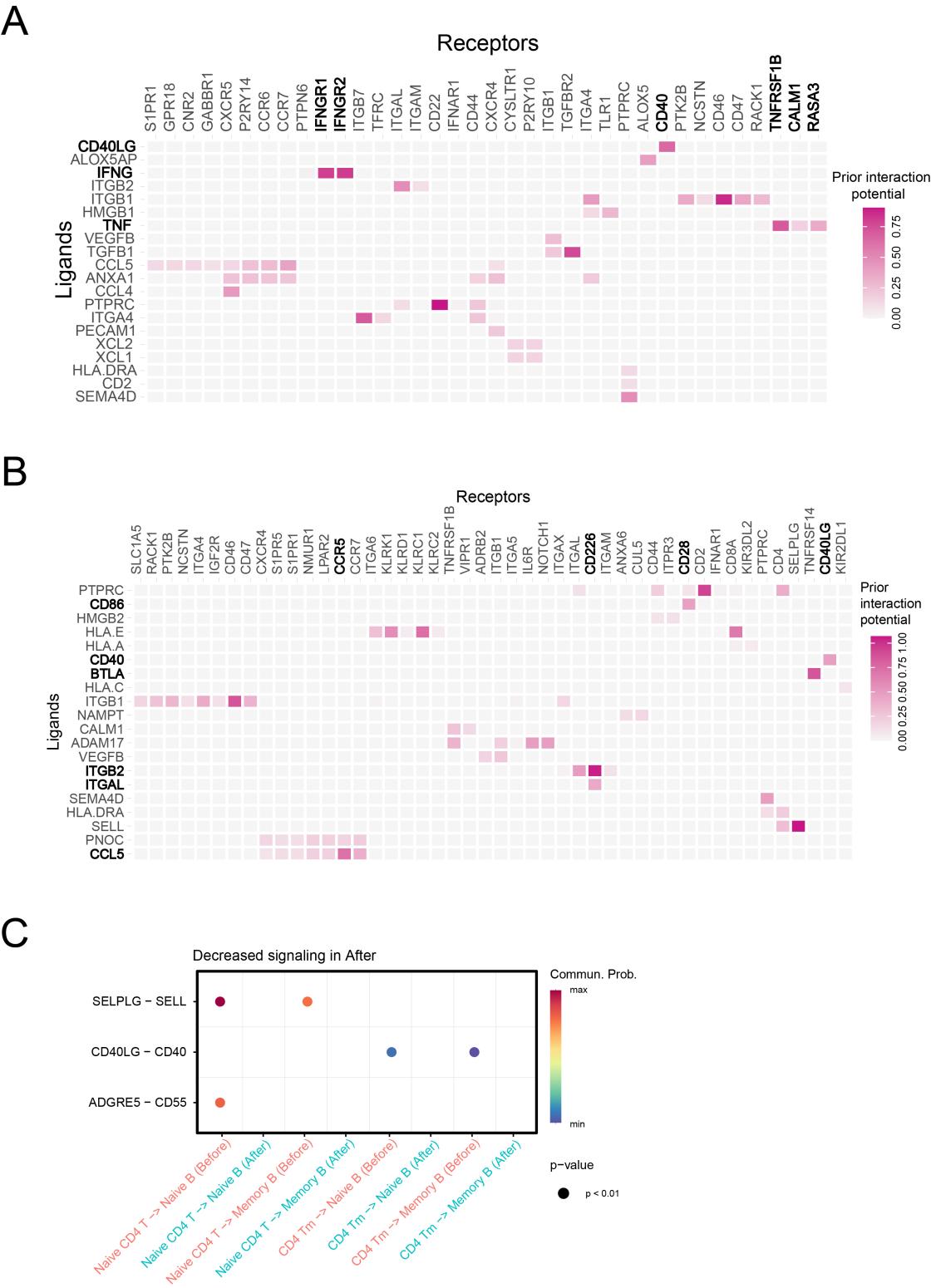
**

1. Signals from T/NK cells to predicted receptors on B cells (pre- and post-FcRn blockade treatment).
2. Signals from B cells to predicted receptors on T/NK cells (pre- and post-FcRn blockade treatment).
3. Dot plot showing the significant changes of indicated cell-to-cell communication pathways between naive/memory CD4^+^ T cells and naive/memory B cells in the paired PBMC samples (pre- and post-FcRn blockade treatment).

**Supplementary Figure 6**


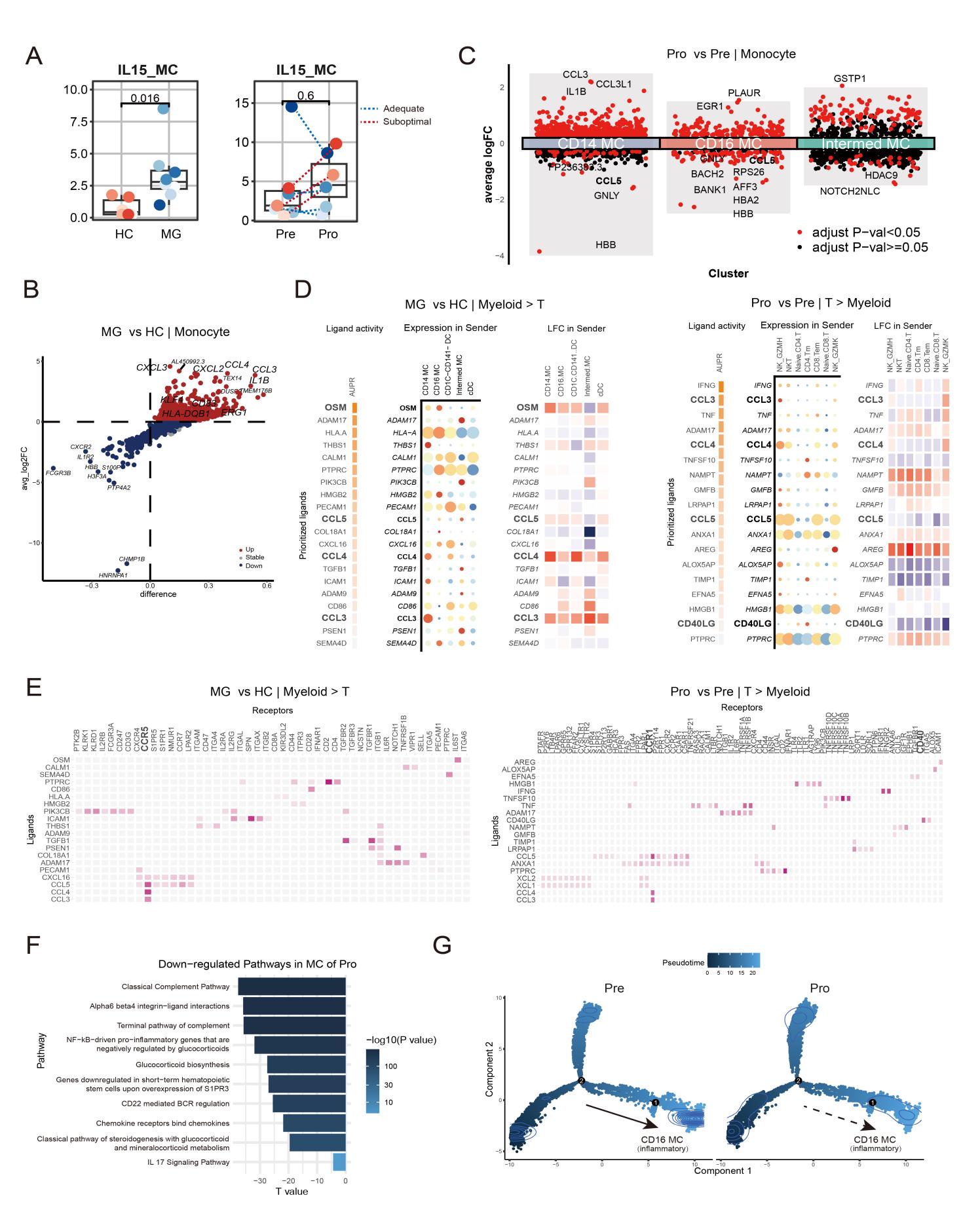


1. Boxplots showing elevated proportions of IL-15⁺ monocytes in patients with gMG compared to healthy controls (HCs), with a subsequent decline observed in adequately responsive patients following FcRn blockade treatment.
2. Volcano plot of differentially expressed genes in monocytes from patients with gMG relative to HCs. Upregulated genes include chemokines (CCL3, CCL4, CCL5, CXCL2, CXCL3), inflammatory mediators (IL1B), antigen presentation and T-cell costimulation markers (HLA-DQB1, CD83), and transcriptional regulators of monocyte activation (EGR1, KLF4).
3. DEGs in monocyte subclusters from post-treatment vs. pre-treatment samples, with reduced expression of CCL5 in CD14⁺ and CD16⁺ monocytes following efgartigimod treatment.
4. NicheNet analysis of intercellular signals from monocyte/DC cells to T cells in HC and gMG groups (left) and signals from T cells to monocyte/DC cells (right) pre- and post-FcRn blockade (ranked by ligand activity).
5. Signals from monocyte/DC cells to predicted receptors on in HC and gMG groups (left) and signals from T cells to predicted receptors on monocyte/DC cells (right) pre- and post-FcRn blockade.
6. Gene set variation analysis (GSVA) of hallmark and curated pathways in monocytes post-treatment, showing downregulation of complement signaling, CD22-mediated BCR regulation, chemokine receptor interaction, and integrin-ligand binding pathways.
7. Pseudotime trajectory analysis of monocyte differentiation states indicating a reduced proportion of CD16⁺ monocytes in terminal proinflammatory states after FcRn blockade.
